# Modified expression of *ZmMYB167* in *Brachypodium distachyon* and *Zea mays* leads to increased cell wall lignin and phenolic content

**DOI:** 10.1038/s41598-019-45225-9

**Published:** 2019-06-19

**Authors:** Rakesh Bhatia, Sue Dalton, Luned A. Roberts, Odin M. Moron-Garcia, Rosario Iacono, Ondrej Kosik, Joe A. Gallagher, Maurice Bosch

**Affiliations:** 1 Institute of Biological, Environmental and Rural Sciences (IBERS), Aberystwyth University, Plas Gogerddan, Aberystwyth, SY23 3EE UK; 2 Rothamsted Research, West Common, Harpenden, Hertfordshire AL5 2JQ UK

**Keywords:** Molecular engineering in plants, Cell wall, Transgenic plants, Molecular engineering in plants

## Abstract

One of the challenges to enable targeted modification of lignocellulosic biomass from grasses for improved biofuel and biochemical production lies within our limited understanding of the transcriptional control of secondary cell wall biosynthesis. Here, we investigated the role of the maize MYB transcription factor ZmMYB167 in secondary cell wall biosynthesis and how modified *ZmMYB167* expression in two distinct grass model species affects plant biomass and growth phenotypes. Heterologous expression of *ZmMYB167* in the C_3_ model system *Brachypodium* led to mild dwarf phenotypes, increased lignin (~7% to 13%) and S-lignin monomer (~11% to 16%) content, elevated concentrations of cell wall-bound *p*-coumaric acid (~15% to 24%) and reduced biomass sugar release (~20%) compared to controls. Overexpression of *ZmMYB167* in the C_4_ model system *Zea mays* increased lignin (~4% to 13%), *p*-coumaric acid (~8% to 52%) and ferulic acid (~13% to 38%) content but did not affect plant growth and development nor biomass recalcitrance. Taken together, modifying *ZmMYB167* expression represents a target to alter lignin and phenolic content in grasses. The *ZmMYB167* expression-induced discrepancies in plant phenotypic and biomass properties between the two grass model systems highlight the challenges and opportunities for MYB transcription factor-based genetic engineering approaches of grass biomass.

## Introduction

Plant biomass represents an abundant, sustainable and renewable resource to meet environmental greenhouse gas reduction obligations set in 2015 at the Paris climate conference of the parties (COP21) and the second phase of the Kyoto Protocol running from 2013 to 2020^1^. Notably, the lignocellulosic component of grasses (*Poaceae*) including sugarcane bagasse, maize stover, rice and wheat straw, along with the dedicated grass biomass crops *Miscanthus* and switchgrass exemplify favoured resources for biofuel production and biorefining into a spectrum of platform chemicals and value‐added bio‐based products^2^. Most of the lignocellulosic biomass, trapped within the secondary cell walls of plants, is comprised of three main polymers, i.e. cellulose (~35 to 45%), hemicellulose (~40 to 50%) and lignin (~10 to 30%)^3^. The parallel-layered sheaths of cellulose microfibrils form the primary structural load-bearing polymer of secondary cell walls. Coating and tethering of cellulose by the grass-specific hemicelluloses glucuronoarabinoxylan (GAX) and β-1,3-1,4 mixed-linkage glucan enhances the mechanical strength of these walls^4^. Lignin, the main non-polysaccharide component of lignocellulosic biomass, further impregnates and coats the cellulose-hemicellulose network in cells undergoing secondary cell wall biosynthesis. This intricate bio-composite provides structural support and integrity, maintains the shape of the cell and strengthens protection against biotic and abiotic factors^5,6^.

Lignin is a complex aromatic and hydrophobic polymer lacking a repeat structure and consisting of hundreds of monolignols, namely, syringyl (S), guaiacyl (G) and *p*-hydroxyphenyls (H) derived from the monolignol-specific pathway^7^. The formation of several chemical bonds including ether, ester, phenyl and covalent bonds between monolignols at multiple positions^8^, confers lignin rigidity, compactness and its ability to fill gaps between and around the cellulose-hemicellulose complexion. The overall abundance, structure, and composition of lignin can vary substantially depending on the biomass feedstock^9^, thus imparting a role to secondary cell wall properties. Grasses also incorporate considerable amounts of ferulic acid (FA) and *p*-coumaric acid (*p*-CA) into the secondary wall, both phenolics derived from the phenylpropanoid pathway. FA, in particular, is involved in cross-linking to GAX and lignin, forming a covalently linked carbohydrate-lignin complex^10^. The functional role of *p*-CA in grass cell walls is less clear, but these phenolics appear to be predominantly attached to lignin through its ester linkages to monolignols, primarily sinapyl alcohol, and are also found to be acylated to GAX^11^.

The relative abundances, cross-linkages, interactions and arrangements of secondary cell wall components within a dense and hydrophobic matrix collectively lead to inherent resistance to cell wall deconstruction, known as biomass recalcitrance^12^. This aspect represents a critical biological as well as a technical barrier for biorefining lignocellulose. Accordingly, studies uncovering the molecular and genetic mechanisms underpinning secondary cell wall biosynthesis and biomass recalcitrance have helped drive traditional plant breeding practices and biotechnological approaches aimed at developing crops for different end-use applications. The well-studied herbaceous feedstock maize (*Zea mays*) represents a powerful and versatile genetic model system and grass crop to address these matters with its completed genome sequence, past breeding success as well as the development of its genetic tools and resources^13^. It is also a member of the highly photosynthetic-efficient C_4_ clade of grasses sharing a close evolutionary phylogeny and degree of gene synteny to the prime biorefining feedstocks *Miscanthus*, switchgrass (*Panicum virgatum*), sorghum (*Sorghum bicolor*), and sugarcane (*Saccharum officinarum*)^14^. Additionally, smaller-genome model grasses such as *Brachypodium distachyon* could facilitate the processes of gene discovery and translational genomics to the genetically more challenging grasses, adding value as a comparative model to increase our knowledge of secondary cell wall biosynthesis and biomass production in grasses^15^.

A sophisticated, extensive and multi-level network of transcription factors (TFs) has emerged over the last decade, controlling the secondary cell wall biosynthesis programme in a highly coordinated and orchestrated fashion^16–18^. Of these, lower network tier MYB (MYELOBLASTOSIS) TFs act as crucial transcriptional regulators of secondary cell wall biosynthesis in several plant species, most frequently studied in the pioneer dicot model plant *Arabidopsis thaliana*^19^. Substantially less published literature is available on bioengineering the next generation of biotechnology grasses using MYB TF-based strategies^20^. For instance, *OsMYB103L* overexpression (OX) and RNA interference (RNAi) in transgenic rice resulted in a ~13% increase and ~15% to 30% decrease in cellulose content respectively^21^. Other work identified *PvMYB4* as a transcriptional repressor of phenylpropanoid biosynthesis and its overexpression in switchgrass resulted in ~50% reduction in lignin and phenolic content, which in turn improved ethanol yields by ~2.5‐fold^22,23^. The overexpression of *SbMYB60*, a transcriptional activator regulating lignin and possibly cellulose/hemicellulose biosynthesis, in sorghum resulted in a ~10% increase in lignin content, leading to a higher energy content of the biomass^24^. In another study, overexpression of *ZmMYB42* was accompanied by a ~8% to 21% reduction in lignin content and ~30% more glucose release in transgenic sugarcane^25^. Overexpression of *ZmMYB42* or *ZmMYB31* in *Arabidopsis* reduced lignin content resulting in dwarfed plants and more enzymatically degradable cell walls^26,27^. Syntelogs of MYB31 and MYB42 across three different grass species (maize, sorghum and rice) were shown to bind to phenylpropanoid gene promoters *in vivo*, although with some subfunctionalisation between the species^28^. *ZmMYB46* or *OsMYB46*, when overexpressed in *Arabidopsis*, were able to activate the entire secondary cell wall biosynthetic programme^29^. *ZmMYB5* and *ZmMYB152* may also play a regulatory role in phenylpropanoid biosynthesis but their function was not evaluated in *planta*^30^. Overall, these transgenic approaches have proven informative to understand the regulatory roles of MYB TFs in secondary cell wall biosynthesis of grasses and underline the potential of MYB TFs for developing advantageous lignocellulosic biomass qualities.

A previously published maize transcriptome analysis revealed several uncharacterised MYBs with potential involvement in the transcriptional regulation of secondary cell wall biosynthesis in grasses^31^. However, their potential to alter lignocellulose properties for improved biomass processing and biochemical production have remained unexplored to date. Here, we utilised two distinct grass model species to investigate the role of a maize MYB TF, designated as *ZmMYB167* (GRMZM2G037650) by the GRASSIUS TF database^32^, in secondary cell wall biosynthesis. Modified expression of *ZmMYB167* in both transgenic *Brachypodium distachyon* and maize plants led to a higher abundance of cell wall lignin and phenolics but with distinct effects on plant growth phenotype and biomass processing properties. These results are informative for TF‐based bioengineering strategies aimed at improving the economic value of bioenergy grasses via carbon-neutral production of biofuels and value-added phenolic crude matter.

## Results

### ZmMYB167 is a potential orthologue of OsMYB42/85, PvMYB42/85A and AtMYB85

**Figure 1:**
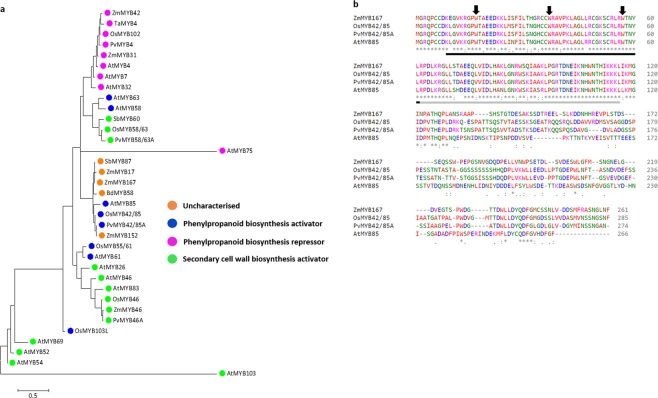
ZmMYB167 is a potential orthologue of phenylpropanoid biosynthesis activators OsMYB42/85, PvMYB42/85A and AtMYB85. (**a**) Maximum likelihood-based phylogenetic analysis of grass and *Arabidopsis* MYB transcriptional regulators of secondary cell wall biosynthesis. Bootstrap support values are from n = 1000 replicates. The scale bar represents the number of substitutions per site. At, *Arabidopsis thaliana*; Os, *Oryza sativa*; Pv, *Panicum virgatum*; Sb, *Sorghum bicolor*; Ta, *Triticum aestivum*; Zm, *Zea mays*. (**b**) Multiple amino acid sequence alignment between ZmMYB167 and characterised MYB transcriptional regulators of phenylpropanoid biosynthesis. The R2 and R3 MYB domains are underlined black and grey respectively below the sequence. The black arrows denote the three regularly spaced tryptophan residues (W), which form the hydrophobic core of the helix-turn-helix structure.

A phylogenetic relationship of known secondary cell wall regulatory MYB TFs was modelled with a maximum likelihood approach to forecast the functional role of the maize MYB TF *ZmMYB167* in secondary cell wall biosynthesis. The phylogenetic tree topology revealed that MYB TFs with similar roles in secondary cell wall biosynthesis clustered together (Fig. 1a). Thus, ZmMYB167 may function similarly to OsMYB42/85, PvMYB42/85A and AtMYB85, which have been demonstrated as transcriptional activators of phenylpropanoid biosynthesis^33–35^. Amino acid sequence alignment showed that the ZmMYB167 protein is 64% and 61% identical to its potential orthologue in rice (OsMYB42/85) and switchgrass (PvMYB42/85A), respectively, and 50% identical with AtMYB85 (Figs 1b and S1). In addition, the phylogenetic analysis revealed ZmMYB17 as a syntelog of ZmMYB167 (Fig. 1a). Motif analysis of ZmMYB167 with the characterised OsMYB42/85, PvMYB42/85A and AtMYB85 proteins further indicated conserved DNA-binding R2 and R3 MYB sites within the N-terminal region and potential functional motifs within the C-terminal region (Fig. S2), the latter usually containing transcriptional activator or repressor activity for the regulation of gene expression^36^. Based on these results coupled with the up-regulation of *ZmMYB167* in a maize internode in which many cells were undergoing secondary cell wall deposition^31^, we hypothesised that ZmMYB167 is an important transcriptional regulator of secondary cell wall biosynthesis in grasses.

### Expression of *ZmMYB167* in *Brachypodium distachyon*

**Figure 2:**
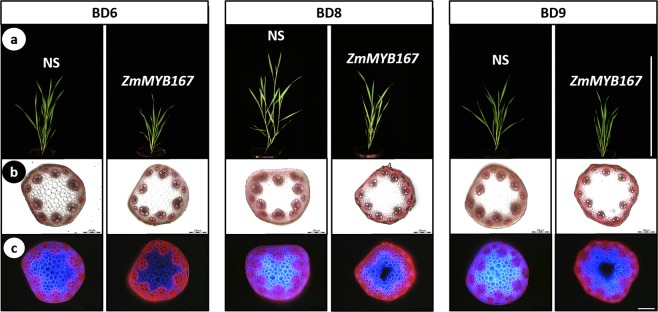
Heterologous *ZmMYB167* expression in *Brachypodium* compromises plant growth and affects lignin deposition in stems. (**a**) Representative phenotype of three independent transgenic *ZmMYB167 Brachypodium* plants 30 days after germination. Scale bar = 30 cm. NS, null-segregant controls. (**b**) Phloroglucinol staining of transverse stem cross-sections of three independent transgenic *ZmMYB167 Brachypodium* plants. Scale bar = 200 μm. (**c**) Double staining with Calcofluor White and phloroglucinol of the same stem cross-sections as shown in (**b**). Scale bar = 200 μm.

**Figure 3:**
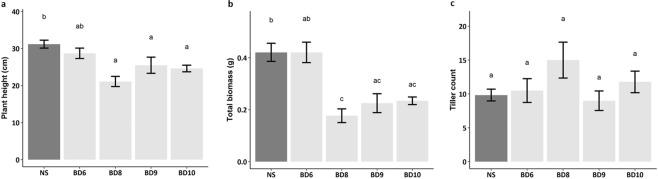
Phenotypic traits from four independent transgenic *ZmMYB167 Brachypodium* plants. (**a**) Plant height measured from base to tip of the inflorescence of the tallest tiller of fully senesced plants. (**b**) Biomass accumulation expressed as total dry above-ground biomass. (**c**) Tiller count recorded as the number of stems per plant. Data are means ± SE of at least four transgenic plants (n ≥ 4). For null-segregant (NS) controls, n = 24. Different letters within each plot indicate significant differences (*P* ≤ 0.05) following a One-way ANOVA with a *post hoc* Tukey test.

**Figure 4:**
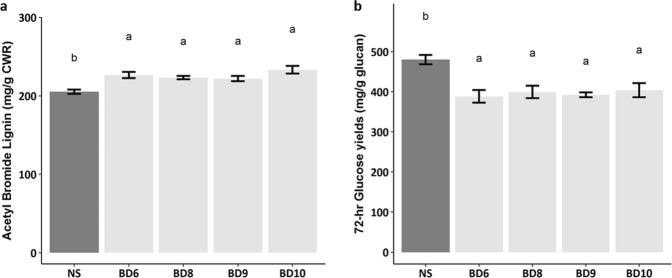
ABSL content and saccharification yields from four independent transgenic *ZmMYB167 Brachypodium* plants. (**a**) ABSL content from fully senesced stems and leaves. (**b**) Glucose hydrolysis yields after 72 hrs. Data are means ± SE of three replicates of at least three transgenic plants (n ≥ 3). For null-segregant (NS) controls, n ≥ 10. Different letters within each plot indicate significant differences (*P* ≤ 0.05) following a One-way ANOVA with a *post hoc* Tukey test.

As a first step to examine the involvement of *ZmMYB167* in the transcriptional regulation of secondary cell wall biosynthesis in grasses *in vivo*, we expressed the *ZmMYB167* gene under control of the constitutive maize ubiquitin promoter (*ZmUbi1*) in *Brachypodium distachyon*. Four independent transgenic lines in the T_1_ generation (named Bd6, Bd8, Bd9 and Bd10) exhibiting heterologous expression of *ZmMYB167* transcripts (Fig. S3a) were evaluated for plant growth and cell wall characteristics. The *ZmMYB167 Brachypodium* expression lines showed reduced plant growth phenotypes 30 days after germination (Fig. 2a). Stem cross-sections from two out of the three randomly selected transgenic *ZmMYB167 Brachypodium* lines stained with phloroglucinol-HCl for lignin demonstrated noticeable increases in lignin deposition particularly in the epidermal cells and cortex (Fig. 2b), possibly induced via the constitutively active *ZmUbi1* promoter. A double staining test with Calcofluor White and phloroglucinol-HCl of the same stem cross-sections showed noticeable staining intensity differences in all three transgenic lines when compared with null-segregant controls (non-transgenic progeny of transgenic parent lines), although the exact cell wall features underpinning these differences remain to be determined (Fig. 2c). Even though transgenic *ZmMYB167 Brachypodium* plants were fertile and produced seeds, plant height was significantly reduced by ~22% and biomass yield by ~43% on average, while tillering was unaffected compared to controls (Fig. 3). Similar results were observed for rice and switchgrass plants overexpressing *OsMYB42/85* and *PvMYB42/85A* respectively which showed a mild dwarf phenotype^34,35^, though growth characteristics were not reported for *AtMYB85*-OX *Arabidopsis* plants^33^. Altered plant growth phenotypes have been reported in several other transgenic grasses because of changes in lignification^22,24^. Indeed, all four *ZmMYB167* transgenic *Brachypodium* lines exhibited increased levels of acetyl bromide soluble lignin (ABSL) content by ~7% to 13% compared to controls (Fig. 4a and Table 1). There were also higher levels of *p*-CA (~15% to 24%) and syringyl (S) lignin monomers (~11% to 16%), lower levels of guaiacyl (G) lignin monomers (~17% to 25%), and a concomitant increase in the S/G ratio (~32% to 53%) in at least three *ZmMYB167* transgenic *Brachypodium* lines (Table 1). While the overall abundance of cell wall polysaccharides remained mostly unchanged (Table 1). Moreover, the glucose yields after 72 hrs of enzymatic hydrolysis of untreated biomass were reduced in all four *ZmMYB167* transgenic *Brachypodium* lines; on average by ~20% compared to controls (Fig. 4b).

**Table 1:** Analysis of cell wall carbohydrates, lignin and phenolic acid content in transgenic *ZmMYB167 Brachypodium* plants.

Genotype	Carbohydrates (mg/g CWR)					Acetyl Bromide	Lignin monomers				Total phenolic acids (mg/g CWR)	
	Glucan	Xylan	Mannan	Arabinan	Galactan	Lignin (mg/g CWR)	H (%)	G (%)	S (%)	S/G ratio	*p*-CA	FA
NS	355.29 ± 3.01^b^	200.91 ± 1.89^b^	1.88 ± 0.04^b^	27.75 ± 0.55^b^	6.98 ± 0.25^b^	207.25 ± 4.44^b^	4.44 ± 0.49^a^	36.61 ± 1.18^a^	58.95 ± 1.40^b^	1.64 ± 0.09^b^	3.48 ± 0.08^b^	6.26 ± 0.42^b^
BD6	362.51 ± 10.59^b^	202.05 ± 6.35^b^	1.97 ± 0.03^b^	28.55 ± 0.68^b^	6.84 ± 0.01^b^	226.62 ± 3.84^a^	3.87 ± 0.06^a^	27.47 ± 0.76^b^	68.66 ± 0.77^a^	2.51 ± 0.10^a^	4.20 ± 0.13^a^	8.74 ± 1.60^a^
BD8	317.27 ± 7.63^a^	186.67 ± 2.98^a^	1.94 ± 0.04^b^	28.93 ± 0.61^b^	6.83 ± 0.15^b^	223.42 ± 2.08^a^	4.80 ± 0.42^a^	27.61 ± 0.45^b^	67.59 ± 0.87^a^	2.45 ± 0.07^a^	4.07 ± 0.23^a^	5.13 ± 0.40^b^
BD9	333.18 ± 10.07^b^	188.63 ± 3.97^b^	1.96 ± 0.04^b^	25.46 ± 0.30^b^	5.69 ± 0.19^b^	222.17 ± 3.32^a^	4.09 ± 0.27^a^	30.53 ± 1.48^ab^	65.38 ± 1.21^ab^	2.16 ± 0.15^ab^	4.00 ± 0.05^b^	6.38 ± 0.84^b^
BD10	332.69 ± 5.03^b^	192.15 ± 5.11^b^	1.92 ± 0.08^b^	26.18 ± 0.93^b^	5.66 ± 0.27^b^	233.35 ± 4.83^a^	4.29 ± 0.05^a^	28.62 ± 1.42^b^	67.09 ± 1.40^a^	2.36 ± 0.17^a^	4.32 ± 0.25^a^	5.06 ± 0.04^a^

Data are means ± SE of three replicates of at least three transgenic plants (n ≥ 3). For null-segregant (NS) controls, n ≥ 10. Different letters within each column indicate significant differences (*P* ≤ 0.05) following a One-way ANOVA with a *post hoc* Tukey test. CWR, cell wall residue. H, *p*-hydroxyphenyl; G, guaiacyl; S, syringyl.

### Overexpression of *ZmMYB167* in *Zea mays*

**Figure 5:**
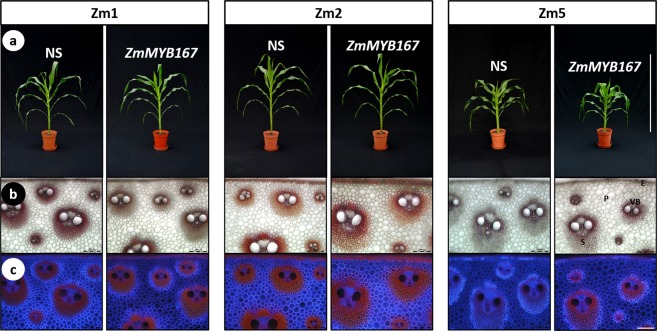
Plant growth and development of three independent *ZmMYB167*-OX maize plants. (**a**) Representative phenotypes of transgenic *ZmMYB167*-OX maize plants at the V12 stage. Scale bar = 1 m. NS, null-segregant controls. (**b**) Phloroglucinol staining of transverse stem cross-sections of three independent transgenic *ZmMYB167*-OX maize plants. E, epidermis; P, parenchyma; S, sclerenchyma; VB, vascular bundle. Scale bar = 200 μm. (**c**) Double staining with Calcofluor White and phloroglucinol of the same stem cross-sections as shown in (**b**). Scale bar = 200 μm.

We next generated independent F_1_ maize progeny from five transformation events (Zm1 to Zm5) harbouring *ZmMYB167* under the control of *ZmUbi1* (Fig. S3b) to study the *ZmMYB167* overexpression (OX) effects in the endogenous model system. Quantitative Real-time PCR analysis verified that *ZmMYB167* expression levels were higher in four transgenic lines (Zm1, Zm2, Zm3 and Zm5), ranging from ~1.5 to 270-fold higher, relative to the respective null-segregant controls for the transgene (Fig. S4). The expression levels of endogenous *ZmMYB167* and the *ZmMYB17* syntelog appeared not to be affected by the overexpression of *ZmMYB167* (Fig. S5). Unlike the phenotypes observed in the C_3_ transgenic *ZmMYB167 Brachypodium* lines, overexpression of *ZmMYB167* in the C_4_ maize lines did not affect plant growth and development (Fig. 5a). Although there was variation in plant height (~124 to 198 cm), flowering time (79 to 87 days), and stem biomass (~24 to 54 g) for the transgenic plants at the vegetative (VT) growth stage, the values for the individual *ZmMYB167* maize lines were not substantially different from their corresponding null-segregant controls originating from the same transformation event (Table S1).

In contrast to the *ZmMYB167 Brachypodium* lines, ectopic lignin deposition was not apparent in phloroglucinol-HCl stained stem cross-sections of the *ZmMYB167*-OX lines when compared to control plants (Fig. 5b). There were also no apparent differences in the double staining with Calcofluor White/phloroglucinol-HCl (Fig. 5c) or the Maüle staining (Fig. S6), as well as in the overall organisation of vascular bundles and fibre cells (Fig. 5b and c). However, ABSL content determined for stem biomass of F_1_ generation plants at the V13 stage was significantly elevated in four of the *ZmMYB167*-OX lines (Zm1, ~8%; Zm2, ~4%; Zm3, ~13% and Zm5, ~7%) when compared to the corresponding control (Table 2). In contrast to the results in *Brachypodium*, the relative percentage of thioacidolysis released S and G lignin monomers, and hence the S/G ratio, were not substantially different between the *ZmMYB167*-OX lines and controls, averaging ~56% and ~42% respectively in both the *ZmMYB167*-OX lines and control plants (Table 2). However, concomitant with an increase in ABSL, the cell walls of these four *ZmMYB167*-OX lines contained significantly higher levels of *p*-CA (~8% to 52%) relative to control samples (Table 2). There were also significantly higher levels of FA (~13% to 38%) in three *ZmMYB167*-OX lines (Table 2). Since both ABSL and total *p*-CA and FA content was higher in at least three *ZmMYB167*-OX lines, we predicted these OX lines to exhibit alterations in Klason lignin. As shown in Table 2, total Klason lignin content was indeed elevated by ~7% to 13% in all the four *ZmMYB167*-OX lines.

**Table 2:** Analysis of lignin and phenolic acid content in stems of transgenic *ZmMYB167* maize plants.

Genotype	Acetyl Bromide	Klason	Lignin monomers				Total phenolic acids (mg/g CWR)	
	Lignin (mg/g CWR)	Lignin (%)	H (%)	G (%)	S (%)	S/G ratio	*p*-CA	FA
NS Zm1	152.95 ± 2.90	14.89 ± 0.19	1.67 ± 0.18	40.65 ± 2.11	57.67 ± 2.29	1.43 ± 0.12	5.37 ± 0.10	4.55 ± 0.13
Zm1	164.43 ± 1.47*	16.14 ± 0.29*	2.02 ± 0.26*	40.34 ± 1.52	57.63 ± 1.78	1.44 ± 0.09	6.98 ± 0.17**	5.54 ± 0.13**
NS Zm2	175.14 ± 0.76	16.28 ± 0.26	1.85 ± 0.34	45.26 ± 4.59	52.89 ± 4.93	1.21 ± 0.21	7.42 ± 0.11	5.55 ± 0.07
Zm2	182.29 ± 0.28**	17.47 ± 0.44**	1.75 ± 0.26	41.49 ± 2.31	56.76 ± 2.57	1.38 ± 0.13	8.00 ± 0.04**	6.25 ± 0.07**
NS Zm3	149.56 ± 2.61	14.08 ± 0.43	2.26 ± 0.25	42.46 ± 1.81	55.28 ± 2.07	1.31 ± 0.10	4.92 ± 0.04	4.18 ± 0.09
Zm3	168.68 ± 1.41**	15.89 ± 0.62**	1.92 ± 0.25**	43.19 ± 2.28	54.89 ± 2.53	1.28 ± 0.12	7.49 ± 0.11**	5.75 ± 0.10**
NS Zm4	168.87 ± 3.60	16.33 ± 0.53	1.62 ± 0.13	45.13 ± 2.02	53.25 ± 2.15	1.19 ± 0.10	5.74 ± 0.08	4.34 ± 0.03
Zm4	175.02 ± 2.39	16.99 ± 0.14	1.63 ± 0.14	43.83 ± 1.79*	54.54 ± 1.92*	1.25 ± 0.09*	5.23 ± 0.18	4.55 ± 0.11
NS Zm5	144.86 ± 1.46	13.29 ± 0.24	1.69 ± 0.17	42.20 ± 1.32	56.10 ± 1.49	1.33 ± 0.08	4.24 ± 0.07	5.23 ± 0.07
Zm5	154.99 ± 2.25**	14.85 ± 0.43*	1.75 ± 0.19	42.51 ± 2.05	55.74 ± 2.25	1.32 ± 0.11	5.19 ± 0.14**	5.21 ± 0.03

Data are means ± SE of three technical replicates from one individual plant per event. Klason lignin is total lignin (acid soluble and acid insoluble lignin) and reported as a percentage of extractives-free CWR, cell wall residue. H, *p*-hydroxyphenyl; G, guaiacyl; S, syringyl. NS, null-segregant. Student’s *t*-test: *P ≤ 0.05; **P ≤ 0.01; ***P ≤ 0.001.

**Figure 6:**
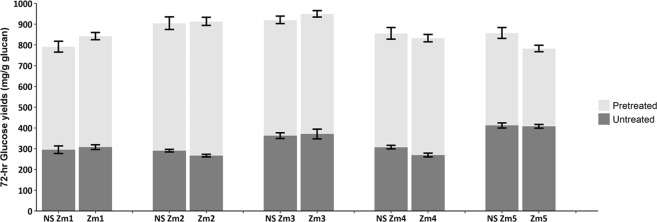
Saccharification yields from untreated and alkaline pre-treated transgenic *ZmMYB167* maize relative to null-segregant (NS) plants. Data are means ± SE of three technical replicates from one individual plant per event. Student’s *t*-test: *P ≤ 0.05; **P ≤ 0.01.

The effects of *ZmMYB167* overexpression on increased lignin and phenolic acids content led us to propose that the stem biomass of these transgenic maize lines may also be less susceptible to enzymatic hydrolysis. Interestingly, none of the *ZmMYB167* transgenic maize lines showed considerable changes in glucose yields (~27% to 41%) after 72 hrs of enzymatic hydrolysis when compared to control samples (Fig. 6). Following a mild pre-treatment with alkaline (0.2 M NaOH), higher glucose yields (~72% to 95%) were obtained, illustrating that the removal of lignin, *p*-CA and FA by this alkaline pre-treatment exposes glucan to enzymatic hydrolysis. However, there were still no significant differences in glucose yields between the *ZmMYB167*-OX lines and controls (Fig. 6).

## Discussion

A thorough understanding of the regulatory mechanisms underlying secondary cell wall biosynthesis is essential to tailor grasses for sustainable bio-based applications. An array of MYB TFs predominantly regulating phenylpropanoid biosynthesis and impacting secondary wall formation have been identified in dicot plants, including *Arabidopsis* and poplar^19,37^. In contrast, relatively few MYB TFs have been directly evaluated in monocots for a regulatory role in secondary cell wall biosynthesis and as a potential target for bioenergy crop improvement^20^. Here, we highlight that modified *ZmMYB167* expression in *Brachypodium* and *Zea mays* can have different implications on plant growth and development as well as cell wall composition and biomass processing efficiency, emphasising both the opportunities and challenges of using MYB TFs as genetic engineering tools.

Phylogenetic analysis revealed that ZmMYB167 is orthologous to the phenylpropanoid biosynthesis activators OsMYB42/85, PvMYB42/85A and AtMYB85 (Fig. 1a), and amino acid sequence analysis of ZmMYB167 indicated that it is a typical R2R3-MYB with conserved R2 and R3 motifs (Figs 1b and S2). A similar candidate gene identification procedure proved effective for identifying TFs involved in secondary cell wall biosynthesis from rice and *Miscanthus*^38,39^. To confirm our hypothesis that *ZmMYB167* regulates lignin biosynthesis, we performed a functional analysis in two different grass model systems, *Brachypodium* and maize. Expression of *ZmMYB167* (heterologous in *Brachypodium* and overexpression in maize) led to increased levels of ABSL in both *Brachypodium* (~7% to 13%) and maize (~4% to 13%) plants compared to control plants (Tables 1 and 2). Such increases in lignin paralleled those of other overexpression approaches using MYB transcriptional activators of lignin biosynthesis. For instance, transgenic *OsMYB42/85*-OX rice plants accumulated a ~4% increase in thioglycolic acid lignin content of leaf blades^34^, whereas transgenic *PvMYB42/85A*-OX switchgrass plants accumulated on average a ~20% increase in ABSL lignin content in whole tillers^35^. Li *et al*.^40^ demonstrated that both ABSL and Klason lignin content significantly increased by ~14% to 28% in stems of transgenic *PtoMYB92*-OX *Populus* plants, while Zhong *et al*.^33^ did not report lignin content for the transgenic *AtMYB85*-OX *Arabidopsis* plants. Hussey *et al*.^41^ postulated that TF overexpression might induce a phenotype within a limited range of overexpression, partly because of the limited amount of protein co-regulators. In our study, the alterations in lignin, *p*-CA and FA content did not correlate well with *ZmMYB167* expression levels in maize plants (Table 2 and Fig. S4), which could imply a TF threshold beyond which there is no further induction of the phenylpropanoid pathway.

The overall abundance of cell wall polysaccharides in *ZmMYB167* transgenic *Brachypodium* lines and *ZmMYB167*-OX maize plants remained largely unchanged (Table 1 and S2), suggesting *ZmMYB167* specifically induces the biosynthesis of lignin and cell wall phenolics without modifying cellulose and hemicellulose biosynthesis. Indeed, Zhong *et al*.^33^ demonstrated *AtMYB85* to specifically induce GUS expression driven by the *4CL1* promoter (lignin), and not for the *CesA8* (cellulose) and *IRX9* (hemicellulose) promoters while Rao *et al*.^35^ showed *PvMYB42/85A* to activate the *COMT* and *F5H* promoters, two major genes in lignin biosynthesis. Future studies will need to determine if ZmMYB167 directly binds to the AC elements ubiquitous in most promoters of lignin biosynthesis genes in monocots. In terms of TFs binding to their own gene promoters^28^, the expression levels of the endogenous *ZmMYB167* and the *ZmMYB17* syntelog did not appear to be affected by the overexpression of *ZmMYB167* in transgenic maize plants (Fig. S5), suggesting that ZmMYB167 auto-regulation (i.e. ZmMYB167 binding to its own gene promoter) and ZmMYB17 cross-regulation is unlikely to have occurred *in planta*.

*ZmMYB167* overexpression in transgenic maize plants had no impact on lignin composition and hence the S/G lignin monomer ratio (Table 2) which can affect biomass recalcitrance^42^, indicative that the carbon flux of the phenylpropanoid pathway towards the biosynthesis of lignin monomers may have remained stable. Among the most distinctive traits of grasses are the phenolics *p*-CA and FA, participating in the composition, cross-linking and structural organisation of secondary cell walls. Concomitant with an increase in lignin content, the biomass of *ZmMYB167*-OX maize plants contained higher levels of cell wall-bound *p*-CA (~8% to 52%) and FA (~13% to 38%) relative to controls (Table 2), whereas the *ZmMYB167* transgenic *Brachypodium* lines exhibited only higher levels of cell wall-bound *p*-CA (~15% to 24%) (Table 1). These elevations in cell wall-bound phenolics are possibly related to the redirection of the metabolic flux along the phenylpropanoid pathway towards these phenolic intermediates. It may be that *p*-CA accumulation occurs in tandem with lignin deposition and could thus be a biochemical indicator to predict lignification^43^. Indeed, our data showed a positive correlation between ABSL and *p*-CA content (r = 0.78), and between ABSL and the *p*-CA: FA ratio (r = 0.71). Together, these results suggest that *ZmMYB167* can fine-tune lignin biosynthesis pathway intermediates and thereby biomass composition.

Interestingly, the *ZmMYB167*-OX maize plants maintained similar growth phenotypes to controls (Table S1), suggesting that increases in the content of cell wall lignin and phenolics did not restrict the expansion of the cell wall during plant growth. In accordance, we also observed no adverse effect on biomass yield in the *ZmMYB167*-OX maize plants (Table S1), which could be relevant and beneficial from an agronomic and biorefining standpoint. Attempts to genetically alter lignin biosynthesis using MYB TFs are frequently accompanied by plant dwarfing or other developmental abnormalities. However, it remains unclear whether such undesirable phenotypic effects directly relate to MYB TF‐induced changes in lignin deposition, hyperaccumulation of phenylpropanoid by-products or an indirect consequence of metabolic spill-over into developmental processes as well as signalling pathways involved in biotic and abiotic stress responses^27,44,45^. Although these TFs represent tools to modify lignin biosynthesis, conducting studies with tissue-specific promoters rather than constitutive promoters could address unintended pleiotropic effects in transgenic plants^46^. In this regard and in contrast to *ZmMYB167*-OX maize plants, heterologous expression of *ZmMYB167* in *Brachypodium* led to a mild dwarf phenotype (Figs 2a and 3a). This phenotypic variation could be a result of different metabolic plasticity, intrinsically variable transcriptional regulatory circuits, changes in spatio-temporal expression of TFs, differences in *cis*-regulatory element composition of genes or protein-protein interactions controlling their distinct tissue organisation and patterning, cell wall formation and growth architecture^47–50^. The adverse phenotypic effects could also be due to the expression of *ZmMYB167* in a heterologous system, with the *Brachypodium* orthologue BdMYB58 (Fig. 1a) showing 65% identity with ZmMYB167 (Fig. S1).

Transgenic approaches targeting MYB TFs have enriched our understanding of the regulatory mechanisms involved in secondary cell wall biosynthesis. Although the ultimate aim of such studies often is to tailor lignocellulose for improved processing and biorefinery application, the impact of transgenic interventions targeting the MYB clade of phenylpropanoid biosynthesis activators on biomass recalcitrance properties has received little attention (Table 3). Even if lignin is commonly referred to as one of the leading factors impeding enzymatic saccharification^51^, the glucose yields of untreated as well as alkaline pre-treated cell wall material from *ZmMYB167*-OX maize plants did not show significant alterations in biomass recalcitrance compared with that of control plants (Fig. 6). This indicates that the higher abundance of cell wall lignin and phenolics did not limit the accessibility of hydrolysing enzymes to matrix polysaccharides nor results in non-productive binding with hydrolysing enzymes and that these secondary wall components alone may not directly reveal the extent of biomass recalcitrance to enzymatic hydrolysis^52–54^. In fact, inhibition of enzymatic hydrolysis by phenolic compounds is more likely related to the presence of different phenolic functional groups^55^. In contrast, the glucose yields of untreated biomass from *ZmMYB167* transgenic *Brachypodium* lines were decreased by ~20% on average (Fig. 4b) and may be explained by the increased S/G ratio (~32% to 53%), a key factor in determining biomass recalcitrance^42,56^ that was not affected in transgenic maize plants (Table 2). Nonetheless, the variable saccharification efficiency observed for the *ZmMYB167* transgenic C_3_ and C_4_ grasses continues to emphasise the complexity of secondary cell wall structures and limited fundamental understanding of how their properties collectively contribute towards biomass recalcitrance.

**Table 3:** Literature related to overexpression of orthologous MYB secondary cell wall-related TFs and biomass recalcitrance properties from maize, rice, switchgrass and *Arabidopsis*.

Clade	Species	TF	Plant phenotype	Lignin content	Lignin monomers	Phenolic content	Carbohydrate content	Biomass recalcitrance	Reference
Monocot	*Zea mays*	ZmMYB167	Normal	~4% to 13% increase in stem	No changes	Increased *p*-CA (~8% to 52%) and FA (~13% to 38%)	No changes	No changes	This study
	*Brachypodium distachyon*	ZmMYB167	Reduced plant height (~22%)	~7% to 13% increase in leaf and stem	Increased S monomers (~11% to 16%) and S/G ratio (~32% to 53%)	Increased *p*-CA (~15% to 24%)	No changes	~20% decrease	This study
	*Oryza sativa*	OsMYB42/85	Reduced plant height (~50%)	~4% increase in leaf blades	NR	NR	NR	NR	Hirano *et al*.^34^
	*Panicum virgatum*	PvMYB42/85 A	Reduced plant height (~36%)	~20% increase in whole tillers	Increased S/G ratio (~4% to 25%)	NR	NR	NR	Rao *et al*.^35^
Dicot	*Arabidopsis thaliana*	AtMYB85	NR	NR	NR	NR	NR	NR	Zhong *et al*.^33^

NR, not reported.

In summary, our findings highlight that *ZmMYB167* expression levels can be modified to increase concentrations of lignin and cell wall-bound phenolics in grasses. We also demonstrate some of the potential challenges associated with MYB TF-based biomass engineering. Considering the economic and ecological importance of several perennial bioenergy grasses, more sophisticated strategies and functional analysis of additional TFs across grasses is needed to improve our understanding of which transcriptional regulatory genes are essential for controlling secondary cell wall biosynthesis and how alterations may impact lignocellulose quality, plant growth and fitness. Such knowledge is vital to help drive traditional plant breeding practices and biotechnological approaches for tailored and improved lignocellulosic biomass production.

## Methods

### Phylogenetic and protein motif analysis

Amino acid sequences were obtained by BLAST search of the NCBI database and analysed for a phylogenetic relationship via alignment using Clustal Omega^57^. Construction of a phylogenetic tree (Maximum likelihood method, Poisson correction model, bootstrap values of 1000) was done using the Molecular Evolutionary Genetics Analysis version 7.0 (MEGA7) program^58^. Protein sequence motifs were identified using the MEME (Multiple Expectation Maximisation for Motif Elicitation) program version 4.12.0^59^.

### Construction of *ZmMYB167* expression cassette and genetic transformation

*ZmMYB167* (GRMZM2G037650; Zm00001d032032) was amplified from maize inbred line B73 cDNA and cloned into the Bb7m24GW destination vector^60,61^, containing the BAR selection marker, for overexpression of *ZmMYB167* in maize. For heterologous expression in *Brachypodium distachyon*, *ZmMYB167* was cloned into the pIPKb002 overexpression vector^62^, containing the hygromycin selection marker. For both vectors, expression of *ZmMYB167* was under the control of the *ZmUbi1* promoter. Overexpression constructs were introduced into *Brachypodium* inbred line Bd21-3 or maize Hi-II hybrid genotype (A188 X B73) by *Agrobacterium*-mediated transformation, using *A*. *tumefaciens* strain EHA108, or particle bombardment (transgenic maize line Zm3 only) as previously described^63–65^. *Brachypodium* and maize plants were selected for characterisation in the T_1_ or F_1_ generation respectively.

### Plant material and growth conditions

Hi-II maize plants were grown in a glasshouse under standard conditions (16 h day; temperature range 22–26 °C; light intensity of 600 μmol m^−2^s^−1^), and T_0_ plants regeneration and F_1_ seed germination were carried out per the Iowa State University “greenhouse care for transgenic maize plants” protocol. Backcrosses and development of F_1_ segregating population were carried out as described by Scott (2013)^66^. All *Brachypodium distachyon* plants were grown in a transgenic glasshouse (16 h day; temperature range 21–23 °C and relative humidity range 40–43%) with a light intensity of 350 μmol m^−2^s^−1^.

### Genomic DNA isolation and PCR analysis

Leaf tissue was harvested from maize (sixth leaf at V8 vegetative stage) and *Brachypodium* plants, frozen in liquid nitrogen and stored at −80 °C until use for genomic DNA (gDNA) isolation using the Qiagen DNAeasy 96 Plant Kit (Qiagen). Following extraction, gDNA concentration and quality were assessed using an Epoch Microplate Spectrophotometer (BioTEK). Gene-specific primers were designed using the NCBI primer designing tool to amplify (i) a DNA fragment covering the *ZmUbi1* promoter region and the transgene-specific region and (ii) the entire DNA fragment of the transgene (Table S3).

### RNA isolation, RT-PCR and Real-time PCR analysis

Total RNA was isolated from −80 °C stored leaf tissue using a combination of Trizol Reagent (Invitrogen) and the Qiagen RNAeasy Plant Mini Kit. RNA quantity was assessed using an Epoch Microplate Spectrophotometer (BioTEK). Quality and integrity of total RNA was evaluated on an agarose gel. cDNA was prepared using the SuperScript III First-Strand Synthesis SuperMix (Invitrogen) with oligo(dT) primers. PCR was performed using the NEB Quick-Load Taq 2X Master Mix (NEB) with the following thermal cycling steps: 95 °C for 2 mins, 35 cycles of 30 sec at 95 °C, 1 min at 60 °C, and 30 sec at 68 °C, final extension 68 °C for 5 mins. RT-PCR products were analysed on 1% agarose gels with *S-adenosylmethionine decarboxylase* or *Peptidase C14* as reference genes for *Brachypodium* and maize respectively.

Real-time PCR was performed on a LightCycler® 480 II system (Roche). Relative quantitative analysis of gene expression was conducted using 1 μl of 2-fold diluted cDNA, 10 μl of SYBR Green I Master Mix, primer pairs and concentrations listed in Tables S4 and S5 and thermal cycling conditions as described above. *Peptidase C14* was used as a reference gene^31^. The Roche Light Cycler® 480 software 1.5 performed relative quantification and primer efficiency-corrected calculations. Data are expressed as means ± SE of at least three independent assays.

### Preparation of cell wall residue

Stem biomass 1 cm above the seventh internode (IN7) from maize plants at vegetative stage 13 (V13) was harvested, prepared using the NREL LAP “Preparation of samples for compositional analysis”^67^ and fractionated to an alcohol insoluble residue (AIR)^68^. Total aboveground leaf and stem biomass of fully senesced *Brachypodium* plants were sampled for preparation of AIR^69^.

### Hydroxycinnamic acids and lignin content

The amounts of total hydroxycinnamic acid derivatives *p*-coumaric acid (*p*-CA) and ferulic acid (FA) was determined as described by Li *et al*.^70^. Acetyl bromide soluble lignin (ABSL) of AIR samples was quantified as described by da Costa *et al*.^68^. Extinction coefficients (g^−1^ L cm^−1^) of *Brachypodium* and maize for the ABSL method of lignin quantification were taken from Barnes and Anderson^71^.

### Thioacidolysis of lignin

Thioacidolysis of AIR was performed as described by Foster *et al*.^69^, with minor modifications. Approximately 2 mg of AIR were transferred into 1.5 ml gas chromatography (GC) vials, and 200 µl of freshly prepared thioacidolysis solution (175 µl dioxane; 20 µl ethanethiol; 5 µl boron trifluoride diethyl etherate) was added per sample. The vial headspace was purged with nitrogen gas, capped immediately, and vials were kept in a heating block at 100 °C for 4 h with gentle mixing every hour. After cooling on ice, 150 μl of 0.4 M NaHCO_3_ was added to neutralise the pH and vial contents were vortexed. For the clean-up, 1 ml of water and 500 µl of ethyl acetate were added to the vials, vortexed and left to stand for a minute to separate the different phases (ethyl acetate on top, water on bottom). 150 μl of the ethyl acetate layer was then transferred into 200 µl GC vials. The ethyl acetate solvent was evaporated, and 200 μl acetone was added and evaporated twice. For the trimethylsilyl (TMS) derivatisation, 30 μl of methoxyamine hydrochloride dissolved in pyridine (20 mg/mL) was added to the vials. After being crimp-capped, the vials were incubated for 15 min at 90 °C in a heating block, de-capped and 20 μl of *N*,*O*-Bis(trimethylsilyl)trifluoroacetamide (BSTFA) was added to derivatise the resulting compounds. The vials were re-capped and incubated for 15 min at 90 °C. GC−MS analysis of thioacidolysis monomers was carried out on a GCMS-QP2010 Plus instrument (Shimadzu Co., Addison, IL) with a Rtx-5MS capillary column (30 m × 0.25 mm × 0.25μm film thickness). Helium was used as the carrier gas. GC-2010 conditions were as follows: Pressure, 76.2 kPa; Total flow, 22.2 ml/min; Column Flow, 0.91 ml/min; Linear Velocity, 36.0 cm/sec; Purge Flow, 3.0 ml/min; Split Ratio, 20.0. The column oven temperature programme was 130 °C, held for 1 min, ramped at 25 °C/min to 225 °C, 3 °C/min to 265 °C and 25 °C/min to 330 °C, and held for 1 min; injection temperature, 280 °C; Split Injector Mode and Linear Velocity Flow Control Mode. Peaks were identified by characteristic mass spectrum ions of 299 m/z, 269 m/z, and 239 m/z for S, G, and H monomers respectively and thioacidolysis monomers quantified using total peak area.

### Structural carbohydrates and Klason lignin

Analysis of cell wall carbohydrate content of *Brachypodium* AIR was performed as described by da Costa *et al*.^54^. Compositional analysis of maize AIR was based on the NREL LAP “Determination of structural carbohydrates and lignin in biomass”^72^. Structural carbohydrates were determined by HPAEC on a Dionex ICS-5000 system (Thermo Fischer Scientific) equipped with a pulsed amperometric detector (PAD) using the Dionex CarboPac SA10 column set at 45 °C and 1 mM KOH as eluent, with an eluent flow rate of 1.5 ml/min and 10 μl injection volume. Monosaccharide chromatograms were analysed and processed using the Chromeleon™ 7.2 Chromatography Data System (CDS) software. The percentage of structural carbohydrates and Klason lignin was reported and calculated on an oven-dry weight and extractives-free basis.

### Enzymatic hydrolysis and alkaline pre-treatment

Low solids enzymatic hydrolysis was carried out based on the NREL LAP “Low solids Enzymatic Saccharification of Lignocellulosic Biomass”^73^ using Accellerase1500 enzymes (DuPont) at a dosage of 60 FPU/g cellulose. Soluble and enzyme-derived sugars were determined by HPAEC using conditions described above. Sugar yields were reported on an oven-dry weight basis, and the correction for hydration/water incorporated upon hydrolysis of cellulose to glucose monomers was applied. AIR samples were also subjected to a mild alkaline pre-treatment at 80 °C for 1 hr using an alkali loading of 0.08 g NaOH per gram of AIR^74^. Following pre-treatment, insoluble solids in pre-treated AIR were determined using the NREL LAP “Determination of Insoluble Solids in Pretreated Biomass Material”^75^. Enzymatic hydrolysis after alkaline pre-treatment was carried out using the NREL LAP “Low Solids Enzymatic Saccharification of Lignocellulosic Biomass”. The data was plotted for glucose yield rather than glucose release to ensure that the results are not biased towards cell walls with higher glucan content, assuming 95% glucan recovery of pre-treated AIR post alkaline pre-treatment^76^.

### Histochemical staining of cellulose and lignin

Maize internode development was assessed and determined using the vegetative and reproductive stage identification system^77^. Maize and *Brachypodium* internode samples were collected in the glasshouse and stored in 70% EtOH at 4 °C until use. The middle portion of internode nine (IN9) from maize and IN1 of the 1st flowering tiller from *Brachypodium* plants were used as sectioning material. Transverse stem cross-sections were freehand-cut with a clean razor blade and stained with 0.01% (w/v) aqueous Calcofluor White (CFW) (Sigma-Aldrich) for 8 min in darkness. The sections were washed with water to rinse away excessive CFW stain and stained with 5% (w/v) phloroglucinol (1,3,5-trihydroxy benzene) (Sigma-Aldrich) in 75% EtOH for 5 min in darkness. The stained sections were transferred onto glass slides and then flooded with drops of 12 N HCl. All stained transverse stem cross sections were mounted on glass slides with 30% glycerol and observed immediately on a Leica LMD6000 microscope.

### Statistical analysis

*Brachypodium* statistical analysis consisted of One-way ANOVA *post-hoc* Tukey test for comparisons of pooled null-segregant controls (inbred line Bd21-3) and independent transgenic *ZmMYB167* plants. The analysis was carried out and plotted using the ggplots package of the R statistics software. Attributing to the maize Hi-II hybrid genotype (A188 X B73), null-segregant controls (plants lacking the *ZmMYB167* transgene) were not pooled and all experimental maize data was subjected to statistical analysis using the Student’s *t*-test performed in Microsoft Office Excel 2016 for comparisons of transgenic *ZmMYB167* plants with corresponding null-segregant controls originating from the same transformation event.

### Accession numbers

BdMYB58 (Bradi3g42430), OsMYB46 (OS12G0515300), OsMYB42/85 (OS09G0532900; Os09g36250), OsMYB102 (Os08g43550), OsMYB103L (OS08G0151300), OsMYB58/63 (OS04G0594100), OsMYB55/61 (OS01G0285300), PvMYB4 (JF299185), PvMYB46A (KT075094), PvMYB42/85A (Pavir.Bb02654), PvMYB58/63A (Pavir.Gb00587), SbMYB60 (SB04G031110), SbMYB87 (Sb07g024970; SORBI_3007G178300), TaMYB4 (JF746995), ZmMYB17 (GRMZM2G138427; Zm00001d053210), ZmMYB31 (GRMZM2G050305; Zm00001d006236), ZmMYB42 (GRMZM2G419239; Zm00001d053220), ZmMYB46 (GRMZM2G052606; Zm00001d023931), ZmMYB152 (GRMZM2G104551; Zm00001d021296), ZmMYB167 (GRMZM2G037650; Zm00001d032032), AtMYB46 (AT5G12870), AtMYB4 (AT4G38620), AtMYB32 (AT4G34990), AtMYB69 (AT4G33450), AtMYB85 (AT4G22680), AtMYB26 (AT3G13890), AtMYB83 (AT3G08500), AtMYB75 (AT2G27190), AtMYB7 (AT2G16720), AtMYB63 (AT1G79180), AtMYB54 (AT1G73410), AtMYB103 (AT1G63910), AtMYB52 (AT1G17950), AtMYB58 (AT1G16490) and AtMYB61 (AT1G09540).

## Supplementary information

Supplementary information

## Data Availability

The datasets generated during and/or analysed during the current study are available from the corresponding author on reasonable request.
