# Supplementary material for: Modified expression of ZmMYB167 in Brachypodium distachyon and Zea mays leads to increased cell wall lignin and phenolic content
Source: Sci Rep. 2019 Jun 19;9:8800. doi: 10.1038/s41598-019-45225-9 (PMC6584667; doi:10.1038/s41598-019-45225-9)
Supplement: Supplementary file 1 — Supplementary information [file 41598_2019_45225_MOESM1_ESM.pdf]

Modified expression of *ZmMYB167* in *Brachypodium distachyon* and *Zea mays* leads to increased cell wall lignin and phenolic content

Rakesh Bhatia, Sue Dalton, Luned A. Roberts, Odin M. Moron-Garcia, Rosario Iacono, Ondrej Kosik, Joe A. Gallagher and Maurice Bosch

Supplementary Information

Figure S1: Pairwise global amino acid sequence alignment between ZmMYB167 and closely related MYB proteins for identities and similarities (%).

Figure S2. DNA binding and potential protein interaction sites of ZmMYB167 with characterised OsMYB42/85, PvMYB42/85A and AtMYB85.

Figure S3. RT-PCR and genomic DNA PCR analysis.

Figure S4. Relative expression levels of *ZmMYB167* in transgenic maize plants.

Figure S5. Semi-quantitative RT-PCR analysis of endogenous *ZmMYB167* and *ZmMYB17* gene of transgenic *ZmMYB167* maize plants.

Figure S6. Mäule staining of lignin in transgenic *ZmMYB167* maize plants.

Figure S7. Full-length uncropped and ungrouped gels for Figure S3 and Figure S5.

Table S1. Agronomic measurements of transgenic *ZmMYB167* maize plants.

Table S2. Structural carbohydrates and lignin in transgenic *ZmMYB167* maize plants.

Table S3. Primers used for DNA sequencing and genomic DNA PCR analysis.

Table S4. Primers used for RT-PCR and Real-time PCR.

Table S5. Information on the standard curves for Real-time PCR.

```
#=======================================
#
# Aligned_sequences: 2
# 1: ZmMYB167
# 2: OsMYB42_85
# Matrix: EBLOSUM62
# Gap_penalty: 10.0
# Extend_penalty: 0.5
#
# Length: 288
# Identity:     183/288 (63.5%)
# Similarity:   206/288 (71.5%)
# Gaps:          30/288 (10.4%)
# Score: 868.5
#
#
#=======================================

ZmMYB167           1 MGRQPCCDKEGVKRGPWTAEEDKKLISFILTHGRCCWRAVPKLAGLLRCG     50
                     |||||||||.|||||||||||||||:|||||:|.||||||||||||||||
OsMYB42_85         1 MGRQPCCDKLGVKRGPWTAEEDKKLMSFILTNGHCCWRAVPKLAGLLRCG     50

ZmMYB167          51 KSCRLRWTNYLRPDLKRGLLSTAEEQLVIDLHAKLGNRWSQIAAKLPGRT    100
                     ||||||||||||||||||||:.||||||||||||||||||:|||||||||
OsMYB42_85        51 KSCRLRWTNYLRPDLKRGLLTDAEEQLVIDLHAKLGNRWSKIAAKLPGRT    100

ZmMYB167         101 DNEIKNHWNTHIKKKLIKMGINPATHQPLANSKAAP---SHSTGTDESAK    147
                     |||||||||||||||||||||:|.||:||...:.:|   |.||.|.||:|
OsMYB42_85       101 DNEIKNHWNTHIKKKLIKMGIDPVTHEPLDRKQESPATTSQSTVTAESSK    150

ZmMYB167         148 SSD-TREELSLKDDNHHREVPLST--DSSEQSSWPEPGSNVG--------    186
                     |.: ||::....||...|::.:|.  ||..:|| ....|..|
OsMYB42_85       151 SGEATRQQSRQLDDAVVRDMSVSAGGDSPPESS-TNTASTAGGSSSSSSS    199

ZmMYB167         187 -DQDPELLVNWPSETDL--SVDESWLGFMSNG--NELGDVEGTS-----P    226
                      .|||  ||.|..|.||  :.||.||.|.::.  :|...:..|.     |
OsMYB42_85       200 HHQDP--LVKWLLEEDLLPTGDEPWLNFTASNDVDEFSSIAATGATPALP    247

ZmMYB167         227 WD--GTTDWLLDYQDFGMCSSNL-VDDSMFRASNGLNF    261
                     ||  .|||||||||||||..|:| ||.||..:|||.||
OsMYB42_85       248 WDVGMTTDWLLDYQDFGMGDSSLVVDASMVNSSNGSNF    285
```

```
#=======================================
#
# Aligned_sequences: 2
# 1: ZmMYB167
# 2: ZmMYB152
# Matrix: EBLOSUM62
# Gap_penalty: 10.0
# Extend_penalty: 0.5
#
# Length: 313
# Identity:     175/313 (55.9%)
# Similarity:   198/313 (63.3%)
# Gaps:          66/313 (21.1%)
# Score: 849.5
#
#
#=======================================

ZmMYB167           1 ------------------------MGRQPCCDKEGVKRGPWTAEEDKKLI     26
                                             |||||||||.||||||||||||:|||
ZmMYB152           1 MGGRRPTYTTRARSAAAALRPAAGMGRQPCCDKLGVKRGPWTAEEDRKLI     50

ZmMYB167          27 SFILTHGRCCWRAVPKLAGLLRCGKSCRLRWTNYLRPDLKRGLLSTAEEQ     76
                     :||||:|.||||||||||||||||||||||||||||||||||||:.||||
ZmMYB152          51 NFILTNGHCCWRAVPKLAGLLRCGKSCRLRWTNYLRPDLKRGLLTDAEEQ    100

ZmMYB167          77 LVIDLHAKLGNRWSQIAAKLPGRTDNEIKNHWNTHIKKKLIKMGINPATH    126
                     :|||||||||||||:||||||||||||||||||||||||||||||:|.||
ZmMYB152         101 VVIDLHAKLGNRWSKIAAKLPGRTDNEIKNHWNTHIKKKLIKMGIDPVTH    150

ZmMYB167         127 QPLANSKAAPSHSTGTDESAKSSD-TREELSLKDDNHHREVP--------    167
                     :|| :.|...|....|.:|.||.: |:|:....||...|:||
ZmMYB152         151 EPL-DRKTTSSGPATTSQSTKSDEATKEQSPQNDDAVIRDVPADGCSPTE    199

ZmMYB167         168 -----LSTDSSEQSSWPEPGSNVGDQDPELLVNWPSETDLSV-DESWLGF    211
                          :||..|..|     |....||||  ||.|..|.:.:. ||:||.|
ZmMYB152         200 SSTNTVSTGGSSSS-----GGGGHDQDP--LVKWLLEEEPATGDEAWLNF    242

ZmMYB167         212 -----------MSNGNELGDVEGTSPWDGTTDWLLDYQDFGMCSSNLVDD    250
                                ::.|.||      .||||.|||||||||||:..|:|||.
ZmMYB152         243 TGSVDVDEFSSIAAGPEL------LPWDGATDWLLDYQDFGLGDSSLVDG    286

ZmMYB167         251 SMF--RASNGLNF    261
                     .|.  .:|||..|
ZmMYB152         287 YMVNNNSSNGAKF    299
```

```
#=======================================
#
# Aligned_sequences: 2
# 1: ZmMYB167
# 2: ZmMYB17
# Matrix: EBLOSUM62
# Gap_penalty: 10.0
# Extend_penalty: 0.5
#
# Length: 264
# Identity:     224/264 (84.8%)
# Similarity:   238/264 (90.2%)
# Gaps:           3/264 ( 1.1%)
# Score: 1191.5
#
#
#=======================================

ZmMYB167           1 MGRQPCCDKEGVKRGPWTAEEDKKLISFILTHGRCCWRAVPKLAGLLRCG     50
                     |||||||||:|||||||||||||||||||||||:||||||||||||||||
ZmMYB17            1 MGRQPCCDKQGVKRGPWTAEEDKKLISFILTHGQCCWRAVPKLAGLLRCG     50

ZmMYB167          51 KSCRLRWTNYLRPDLKRGLLSTAEEQLVIDLHAKLGNRWSQIAAKLPGRT    100
                     |||||||||||||||||||||||||||||||||.||||||:|||||||||
ZmMYB17           51 KSCRLRWTNYLRPDLKRGLLSTAEEQLVIDLHAMLGNRWSKIAAKLPGRT    100

ZmMYB167         101 DNEIKNHWNTHIKKKLIKMGINPATHQPLANSK-AAPSHSTGTDESAKSS    149
                     ||||||||||||||||:||||:||||||||||| ||.|.||.||||||:|
ZmMYB17          101 DNEIKNHWNTHIKKKLVKMGIDPATHQPLANSKEAASSQSTVTDESAKTS    150

ZmMYB167         150 DTREELSLKDDNHHREVPLSTDSSEQSSWPE--PGSNVGDQDPELLVNWP    197
                     ::|||||||:|...||.||||||||||||||  .||||.||.||||.||.
ZmMYB17          151 NSREELSLKNDGSRREAPLSTDSSEQSSWPESGSGSNVCDQGPELLENWL    200

ZmMYB167         198 SETDLSVDESWLGFMSNGNELGDVEGTSPWDGTTDWLLDYQDFGMCSSNL    247
                     |||.||:||.||.|.|..:|||:||||.|||||||||||||||||||||.
ZmMYB17          201 SETGLSMDEPWLDFTSRNDELGNVEGTLPWDGTTDWLLDYQDFGMCSSNS    250

ZmMYB167         248 VDDSMFRASNGLNF    261
                     ||:|:|.||||.||
ZmMYB17          251 VDNSVFHASNGSNF    264
```

```
#=======================================
#
# Aligned_sequences: 2
# 1: ZmMYB167
# 2: SbMYB87
# Matrix: EBLOSUM62
# Gap_penalty: 10.0
# Extend_penalty: 0.5
#
# Length: 261
# Identity:     225/261 (86.2%)
# Similarity:   237/261 (90.8%)
# Gaps:           4/261 ( 1.5%)
# Score: 1207.0
#
#
#=======================================

ZmMYB167           1 MGRQPCCDKEGVKRGPWTAEEDKKLISFILTHGRCCWRAVPKLAGLLRCG     50
                     |||||||||:||||||||||||||||||||||||||||||||||||||||
SbMYB87            1 MGRQPCCDKQGVKRGPWTAEEDKKLISFILTHGRCCWRAVPKLAGLLRCG     50

ZmMYB167          51 KSCRLRWTNYLRPDLKRGLLSTAEEQLVIDLHAKLGNRWSQIAAKLPGRT    100
                     ||||||||||||||||||||||||||||||||||||||||:|||||||||
SbMYB87           51 KSCRLRWTNYLRPDLKRGLLSTAEEQLVIDLHAKLGNRWSKIAAKLPGRT    100

ZmMYB167         101 DNEIKNHWNTHIKKKLIKMGINPATHQPLANSKAAPSHSTGTDESAKSSD    150
                     ||||||||||||||||||||::|||||||:||.|..|.|||.||||||||
SbMYB87          101 DNEIKNHWNTHIKKKLIKMGLDPATHQPLSNSNATSSQSTVTDESAKSSD    150

ZmMYB167         151 TREELSLKDDNHHREVPLSTDSSEQSSWPEPGSNVGDQDPELLVNWPSET    200
                     .|||||||||: ||||||||||||||||||..:|..|||||||||||
SbMYB87          151 FREELSLKDDS-HREVPLSTDSSEQSSWPESSTNGCDQDPELLVNWP---    196

ZmMYB167         201 DLSVDESWLGFMSNGNELGDVEGTSPWDGTTDWLLDYQDFGMCSSNLVDD    250
                     ||.:||.||.|||:.:|||:|||..|||||||||||||||||||||.||:
SbMYB87          197 DLLMDEPWLNFMSSNDELGNVEGALPWDGTTDWLLDYQDFGMCSSNSVDN    246

ZmMYB167         251 SMFRASNGLNF    261
                     |.|.||||.||
SbMYB87          247 STFHASNGSNF    257
```

```
#=======================================
#
# Aligned_sequences: 2
# 1: ZmMYB167
# 2: AtMYB85
# Matrix: EBLOSUM62
# Gap_penalty: 10.0
# Extend_penalty: 0.5
#
# Length: 302
# Identity:     151/302 (50.0%)
# Similarity:   173/302 (57.3%)
# Gaps:          77/302 (25.5%)
# Score: 681.5
#
#
#=======================================

ZmMYB167           1 MGRQPCCDKEGVKRGPWTAEEDKKLISFILTHGRCCWRAVPKLAGLLRCG     50
                     |||||||||.|||:||||.|||||||:||||:|.|||||:||||||.|||
AtMYB85            1 MGRQPCCDKLGVKKGPWTVEEDKKLINFILTNGHCCWRALPKLAGLRRCG     50

ZmMYB167          51 KSCRLRWTNYLRPDLKRGLLSTAEEQLVIDLHAKLGNRWSQIAAKLPGRT    100
                     |||||||||||||||||||||..||||||||||.|||:||:||::|||||
AtMYB85           51 KSCRLRWTNYLRPDLKRGLLSHDEEQLVIDLHANLGNKWSKIASRLPGRT    100

ZmMYB167         101 DNEIKNHWNTHIKKKLIKMGINPATHQPL-------ANSKAAPSHS----    139
                     ||||||||||||||||:||||:|.|||||       .|||..||:.
AtMYB85          101 DNEIKNHWNTHIKKKLLKMGIDPMTHQPLNQEPSNIDNSKTIPSNPDDVS    150

ZmMYB167         140 ---------------TGTDESAKSSDTREELSLKDDNHHREVPLSTDSSE    174
                                    |.|:|.:.|:.|.:..|:.::||..:
AtMYB85          151 VEPKTTNTKYVEISVTTTEEESSSTVTDQNSSMDNENHLID---------    191

ZmMYB167         175 QSSWPEPGSNVGDQDPELLVNWPSETDLSVDE-SWLGFMSN---GNELGD    220
                              |:.|.|......|..||  :.|| ||..  ||   |..|.|
AtMYB85          192 ---------NIYDDDELFSYLWSDET--TKDEASWSD--SNFGVGGTLYD    228

ZmMYB167         221 --VEGTSP----WD----GTTDWLLDY-QDFGMCSSNLVDDSMFRASNGL    259
                       :.|...    |.    ......||| ||||      |.|..|
AtMYB85          229 HNISGADADFPIWSPERINDEKMFLDYCQDFG------VHDFGF------    266

ZmMYB167         260 NF    261

AtMYB85          267 --    266
```

```
#=======================================
#
# Aligned_sequences: 2
# 1: ZmMYB167
# 2: PvMYB42_85A
# Matrix: EBLOSUM62
# Gap_penalty: 10.0
# Extend_penalty: 0.5
#
# Length: 277
# Identity:     170/277 (61.4%)
# Similarity:   196/277 (70.8%)
# Gaps:          19/277 ( 6.9%)
# Score: 852.0
#
#
#=======================================

ZmMYB167           1 MGRQPCCDKEGVKRGPWTAEEDKKLISFILTHGRCCWRAVPKLAGLLRCG     50
                     |||||||||.||||||||||||:|||||||::|.||||||||||||||||
PvMYB42_85A        1 MGRQPCCDKLGVKRGPWTAEEDRKLISFILSNGHCCWRAVPKLAGLLRCG     50

ZmMYB167          51 KSCRLRWTNYLRPDLKRGLLSTAEEQLVIDLHAKLGNRWSQIAAKLPGRT    100
                     ||||||||||||||||||||:.||||:|||||||||||||:|||||||||
PvMYB42_85A       51 KSCRLRWTNYLRPDLKRGLLTDAEEQVVIDLHAKLGNRWSKIAAKLPGRT    100

ZmMYB167         101 DNEIKNHWNTHIKKKLIKMGINPATHQPL----ANSKAAPSHSTGTDESA    146
                     |||||||||||||||||||||:|.||:||    :||.|..|.|..|.:|.
PvMYB42_85A      101 DNEIKNHWNTHIKKKLIKMGIDPVTHEPLDRKTSNSPATTSQSVVTADST    150

ZmMYB167         147 KSSDTREELSLKDD---NHHREVPLSTDSSEQSSWPEPGSNVG---DQDP    190
                     ||.:..::.|.:.|   :...:....|:||..::....|.::.   ||||
PvMYB42_85A      151 KSDEATKQQSPQSDAVGDVLADGSSPTESSATNTTVSTGGSISSSHDQDP    200

ZmMYB167         191 ELLVNWPSETDLSV-DESWLGFMSNGNELGDVEGTS-----PWDGTTDWL    234
                       ||.|..|.|... ||.||.|..:....|:....:     ||||.||||
PvMYB42_85A      201 --LVKWLLEVDPPTGDEPWLNFTGSVEVDGEFSSIAAGPELPWDGATDWL    248

ZmMYB167         235 LDYQDFGMCSSNLVDDSMFRASNGLNF    261
                     |||||||:....|||..|..:|||.|
PvMYB42_85A      249 LDYQDFGLGDLGLVDGYMINSSNGAN-    274
```

```
#=======================================
#
# Aligned_sequences: 2
# 1: ZmMYB167
# 2: BdMYB58
# Matrix: EBLOSUM62
# Gap_penalty: 10.0
# Extend_penalty: 0.5
#
# Length: 274
# Identity:     179/274 (65.3%)
# Similarity:   196/274 (71.5%)
# Gaps:          19/274 ( 6.9%)
# Score: 904.0
#
#
#=======================================

ZmMYB167           1 MGRQPCCDKEGVKRGPWTAEEDKKLISFILTHGRCCWRAVPKLAGLLRCG     50
                     |||||||||:||||||||||||.||:||||||||||||||||||||||||
BdMYB58            1 MGRQPCCDKQGVKRGPWTAEEDNKLVSFILTHGRCCWRAVPKLAGLLRCG     50

ZmMYB167          51 KSCRLRWTNYLRPDLKRGLLSTAEEQLVIDLHAKLGNRWSQIAAKLPGRT    100
                     ||||||||||||||||||||:..||:||:|||||||||||:|||||||||
BdMYB58           51 KSCRLRWTNYLRPDLKRGLLTADEEKLVVDLHAKLGNRWSKIAAKLPGRT    100

ZmMYB167         101 DNEIKNHWNTHIKKKLIKMGINPATHQPLANSKAA-PSHSTGTDESAKSS    149
                     |||||||||||||||||||||:|||||||||:||| |..:|....|.|||
BdMYB58          101 DNEIKNHWNTHIKKKLIKMGIDPATHQPLANTKAASPIGTTSQSTSFKSS    150

ZmMYB167         150 DTREELSLKDDNHHREVPLSTDSSEQSSWPEPGSNVGDQDPELLVNWPSE    199
                     ||..:|..| ..|...|...|||||.||     .|..|.|.:||.|...|
BdMYB58          151 DTVNQLDSK-GTHGEGVSRPTDSSEHSS-----RNTSDDDQDLLANLLLE    194

ZmMYB167         200 TDLSVDESWLGF-MSNGNELGDVEGTSPWDGTTDWLLDYQDFGMCSSNLV    248
                     .:|.|||.||.| .||.:|...:.|....||.|||||||||.||..|:|:
BdMYB58          195 EELPVDEPWLNFESSNDDEFSSIIGQLLCDGPTDWLLDYQDVGMGGSSLI    244

ZmMYB167         249 DDSMFRASNGLNF-----------    261
                     |......|:.|..
BdMYB58          245 DQDFGSPSSNLTEGAVRSSEESKF    268
```

Figure S1. Pairwise global amino acid sequence alignment between ZmMYB167 and closely related MYB proteins for identities and similarities (%). Alignments were made using EMBOSS Needle (EMBL-EBI).

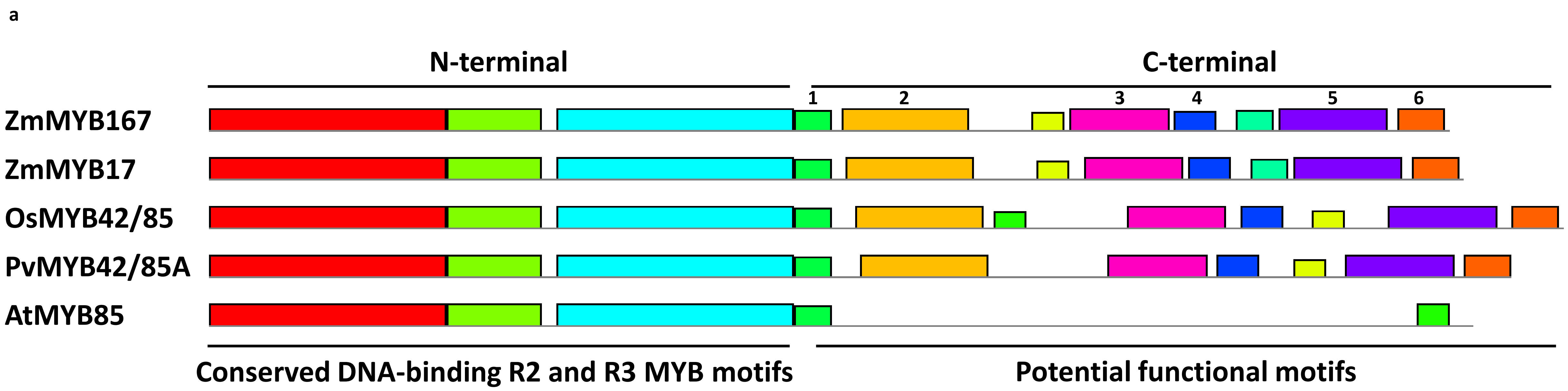

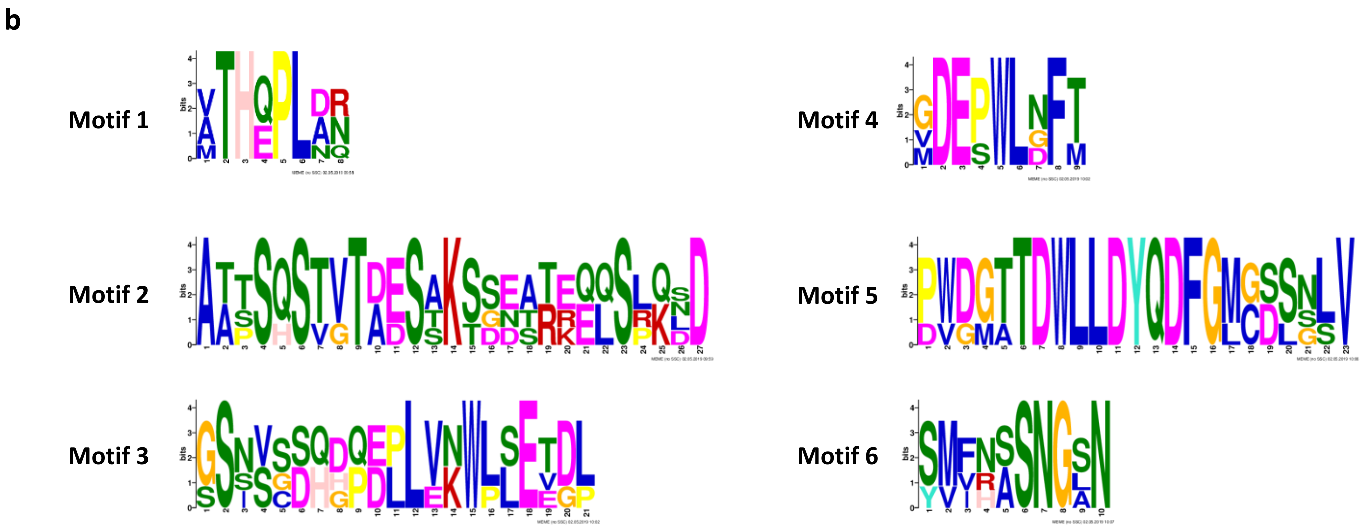

Figure S2. DNA binding and potential protein interaction sites of ZmMYB167 with characterised OsMYB42/85, PvMYB42/85A and AtMYB85. (a) Each coloured block shows the position of an individual motif. The height of a block gives an indication of the significance of the site with taller blocks being more significant. At, *Arabidopsis thaliana*; Os, *Oryza sativa*; Pv, *Panicum virgatum*; Zm, *Zea mays*. (b) Conserved and most statistically significant C-terminal motifs identified for ZmMYB167 and ZmMYB17 with characterised OsMYB42/85, PvMYB42/85A and AtMYB85 proteins, suggesting possible functional similarity. The bit score (y-axis) indicates the relative frequency of the corresponding amino acid.

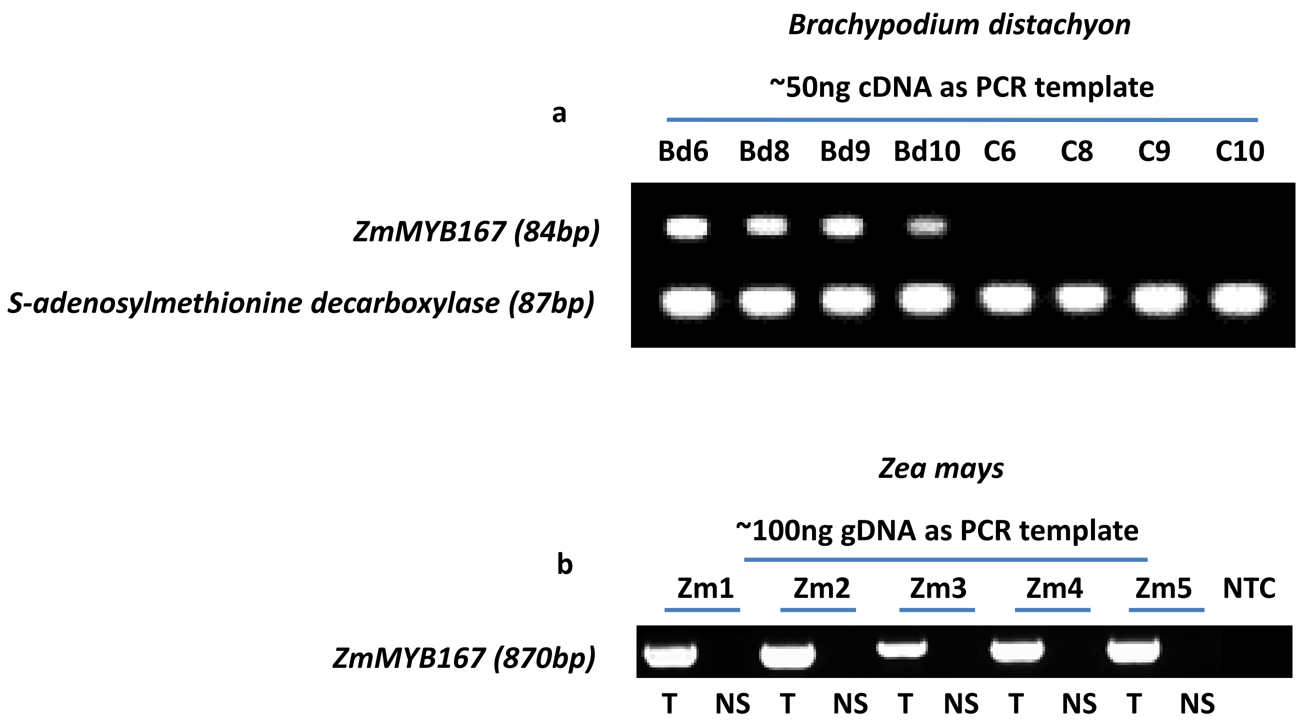

Figure S3. RT-PCR and genomic DNA PCR analysis. (a) RT-PCR analysis of *ZmMYB167* transgenic *Brachypodium* using *ZmMYB167* and *S-adenosylmethionine decarboxylase* gene-specific primers. Bd6 to Bd10 indicate the transgenic lines while C6 to C10 indicate the corresponding T_1 null-segregating control plants. (b) genomic DNA PCR analysis of *ZmMYB167* transgenic and corresponding null-segregant plants using primers covering the *ZmUbi1* promoter region and the *ZmMYB167* transgene-specific region. T, transgenic line; NS, null-segregant control; NTC, no template control. The full-length gels for Figure S3 are presented in Figure S7a-c.

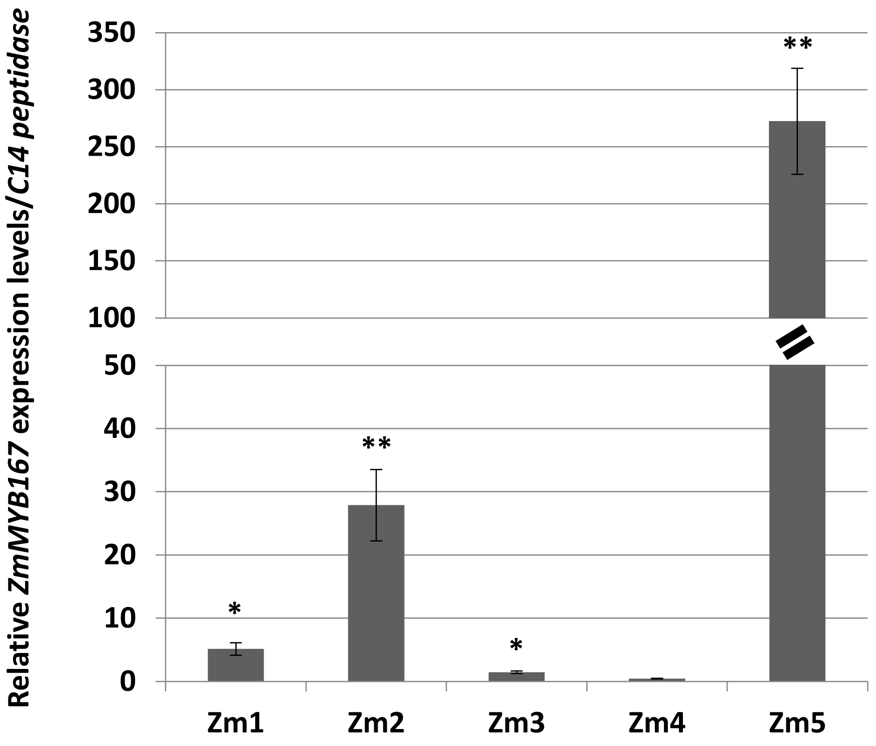

Genotype	Fold change
**NS Zm1**	1.00 ± 0.25
**Zm1**	5.13 ± 0.98*
**NS Zm2**	1.00 ± 0.10
**Zm2**	27.87 ± 5.65**
**NS Zm3**	1.00 ± 0.12
**Zm3**	1.45 ± 0.22*
**NS Zm4**	1.00 ± 0.26
**Zm4**	0.43 ± 0.07
**NS Zm5**	1.00 ± 0.09
**Zm5**	271.04 ± 46.41**

Figure S4. Relative expression levels of *ZmMYB167* in transgenic maize plants. *Peptidase C14* was used as a reference gene. Data are means ± SE of three technical replicates from one individual plant per event. The expression level of *ZmMYB167* in the relative corresponding null-segregant (NS) plant was set to 1. Student's *t*-test: *P ≤0.05; **P ≤0.01.

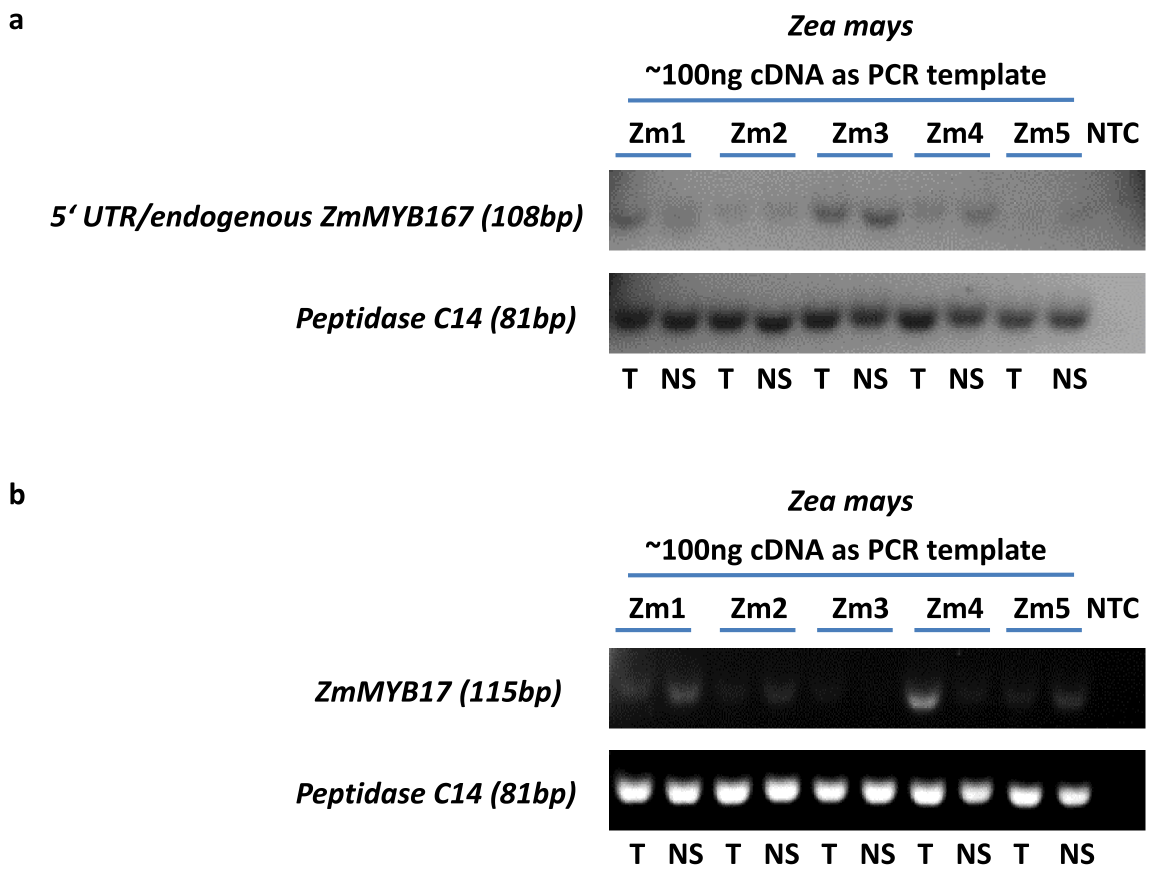

Figure S5. Semi-quantitative RT-PCR analysis of *endogenous ZmMYB167* and *ZmMYB17 gene* of transgenic *ZmMYB167* maize plants. PCR amplification was programmed as follows: 95°C for 30s, 60°C for 1min and 68°C for 30s. After (a) 30 cycles and (b) 35 cycles of amplification, PCR reactions were further incubated at 68°C for 5min and then chilled at 4°C. *Peptidase C14* was used as a reference gene. T, transgenic line; NS, null-segregant control; NTC, no template control. Image (a) was colour inverted for better visibility. The full-length gels for Figure S5 are presented in Figure S7d-f.

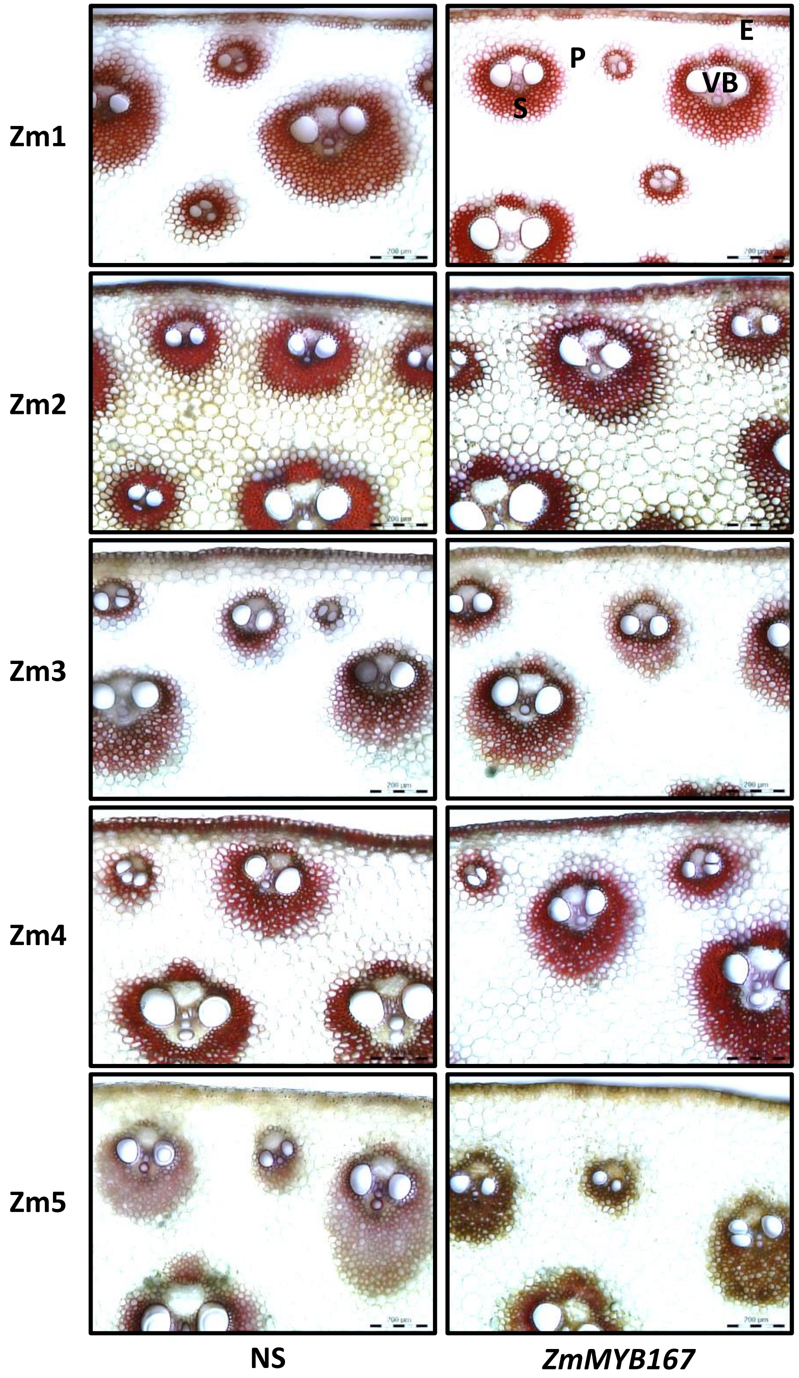

Figure S6. Maüle staining of lignin in transgenic *ZmMYB167* maize plants. Hand-cut transverse stem cross-sections of internode nine imaged under bright field. Stem sections sampled from plants at V13 stage. Mäule staining stains S lignin monomers red and G lignin monomers orange-brown. NS, null-segregant; E, epidermis; P, parenchyma; S, sclerenchyma; VB, vascular bundle. Scale bar =200µm.

ZmMYB167 transcript

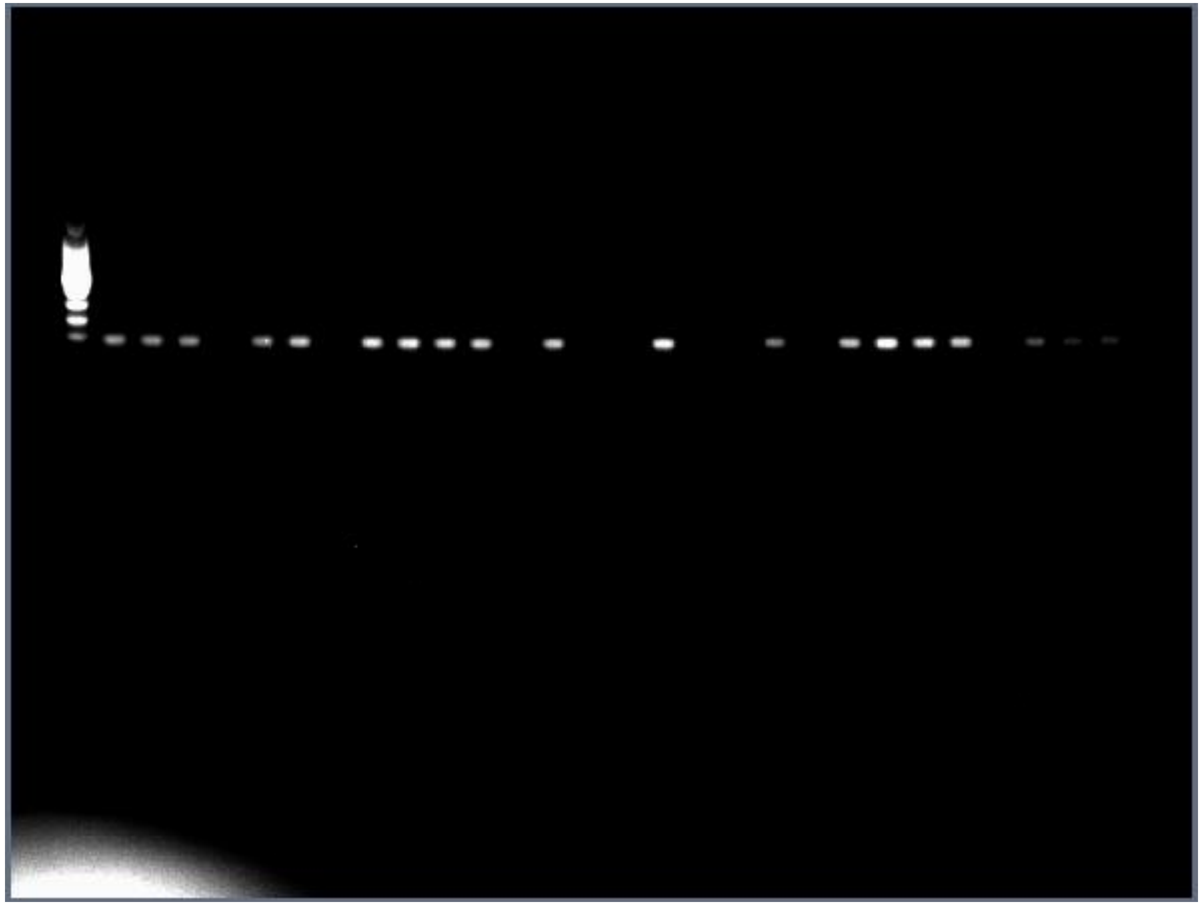

PCR Lane No	T1 ZmMYB167 Brachy plant	cDNA template (~50ng/µl)	NEB Quick-Load Taq 2X Master Mix (µl)	Forward Primer 5µM (µl)	Reverse Primer 5µM (µl)	DEPC water (µl)	Forward Primer	Reverse Primer	Amplicon size (bp)
1	100bp molecular marker								
2	BD10 VIII	1.0	12.5	0.5	0.5	10.5	MB167	MB168	84
3	BD10 IV	1.0	12.5	0.5	0.5	10.5	MB167	MB168	84
4	BD10 XIII	1.0	12.5	0.5	0.5	10.5	MB167	MB168	84
5	BD10 XVI*	1.0	12.5	0.5	0.5	10.5	MB167	MB168	84
6	BD10 II	1.0	12.5	0.5	0.5	10.5	MB167	MB168	84
7	BD10 VII	1.0	12.5	0.5	0.5	10.5	MB167	MB168	84
8	BD9 I*	1.0	12.5	0.5	0.5	10.5	MB167	MB168	84
9	BD9 XVIII	1.0	12.5	0.5	0.5	10.5	MB167	MB168	84
10	BD9 VII	1.0	12.5	0.5	0.5	10.5	MB167	MB168	84
11	BD9 XIX	1.0	12.5	0.5	0.5	10.5	MB167	MB168	84
12	BD9 XII	1.0	12.5	0.5	0.5	10.5	MB167	MB168	84
13	BD9 IX*	1.0	12.5	0.5	0.5	10.5	MB167	MB168	84
14	BD9 XIV	1.0	12.5	0.5	0.5	10.5	MB167	MB168	84
15	BD6 XI*	1.0	12.5	0.5	0.5	10.5	MB167	MB168	84
16	BD6 VI*	1.0	12.5	0.5	0.5	10.5	MB167	MB168	84
17	BD6 X	1.0	12.5	0.5	0.5	10.5	MB167	MB168	84
18	BD6 XV*	1.0	12.5	0.5	0.5	10.5	MB167	MB168	84
19	BD6 XIV*	1.0	12.5	0.5	0.5	10.5	MB167	MB168	84
20	BD8 IX	1.0	12.5	0.5	0.5	10.5	MB167	MB168	84
21	BD8 II*	1.0	12.5	0.5	0.5	10.5	MB167	MB168	84
22	BD8 III	1.0	12.5	0.5	0.5	10.5	MB167	MB168	84
23	BD7 XVIII	1.0	12.5	0.5	0.5	10.5	MB167	MB168	84
24	BD7 I	1.0	12.5	0.5	0.5	10.5	MB167	MB168	84
25	BD7 II	1.0	12.5	0.5	0.5	10.5	MB167	MB168	84
26	BD1 VII*	1.0	12.5	0.5	0.5	10.5	MB167	MB168	84
27	BD1 III	1.0	12.5	0.5	0.5	10.5	MB167	MB168	84
28	BD1 XV	1.0	12.5	0.5	0.5	10.5	MB167	MB168	84
29	BD1 X	1.0	12.5	0.5	0.5	10.5	MB167	MB168	84
30	NTC	0.0	12.5	0.5	0.5	11.5	MB167	MB168	84

Thermal Cycling conditions:	Initial Denaturation	Denaturation	Annealing	Extension	Final Extension
	95C for 2mins	95C for 0.5mins	60C for 1min	68C for 0.5mins	68C for 5mins
			30 cycles		

Figure S7a. Full length gel for the results shown in **Figure S3a** of the RT-PCR analysis of *ZmMYB167* transgenic *Brachypodium* using *ZmMYB167* gene-specific primers. * Denotes null-segregant (NS). NTC, no template control.

S-adenosylmethionine decarboxylase transcript

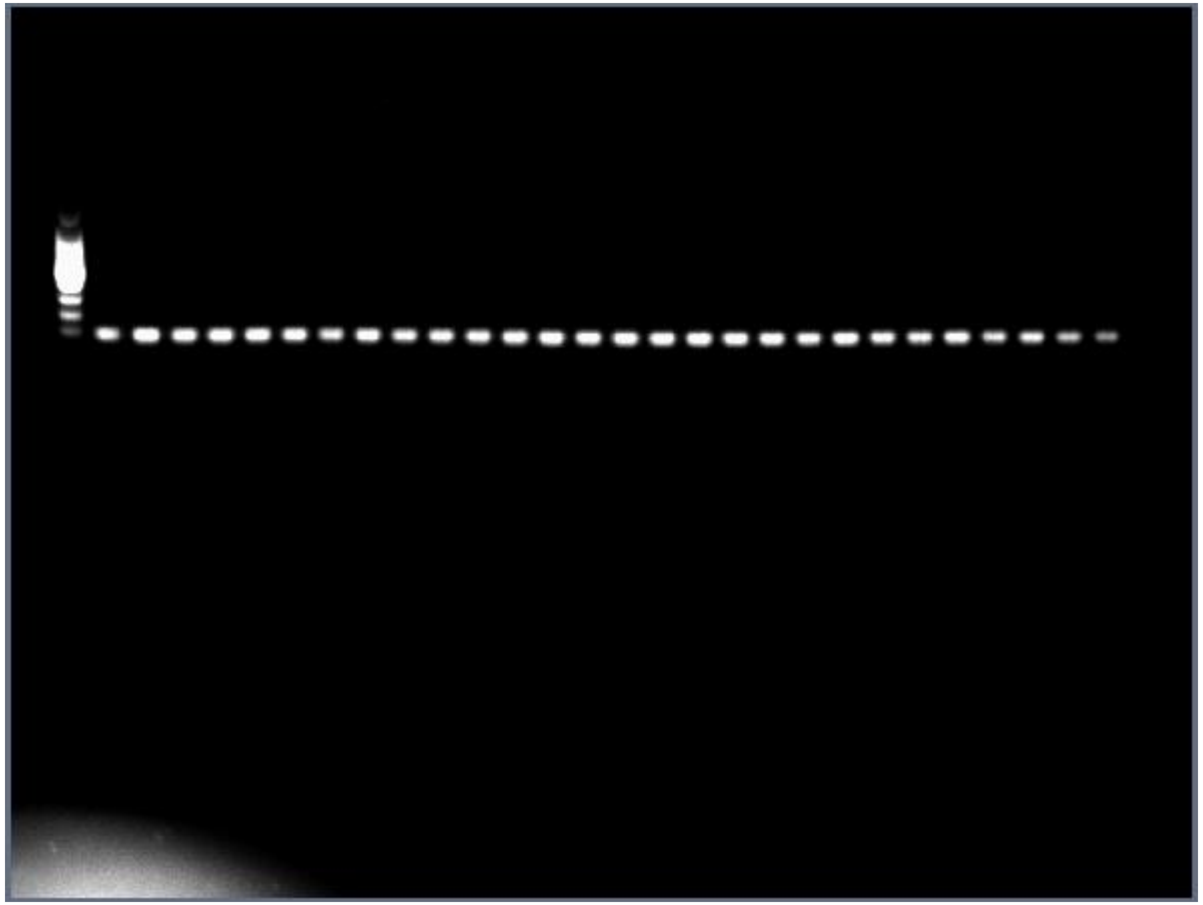

PCR Lane No	T1 ZmMYB167 Brachy plant	cDNA template (~50ng/µl)	NEB Quick-Load Taq 2X Master Mix (µl)	Forward Primer 5µM (µl)	Reverse Primer 5µM (µl)	DEPC water (µl)	Forward Primer	Reverse Primer	Amplicon size (bp)
1	100bp molecular marker								
2	BD10 VIII	1.0	12.5	0.5	0.5	10.5	LF72	LF73	87
3	BD10 IV	1.0	12.5	0.5	0.5	10.5	LF72	LF73	87
4	BD10 XIII	1.0	12.5	0.5	0.5	10.5	LF72	LF73	87
5	BD10 XVI*	1.0	12.5	0.5	0.5	10.5	LF72	LF73	87
6	BD10 II	1.0	12.5	0.5	0.5	10.5	LF72	LF73	87
7	BD10 VII	1.0	12.5	0.5	0.5	10.5	LF72	LF73	87
8	BD9 I*	1.0	12.5	0.5	0.5	10.5	LF72	LF73	87
9	BD9 XVIII	1.0	12.5	0.5	0.5	10.5	LF72	LF73	87
10	BD9 VII	1.0	12.5	0.5	0.5	10.5	LF72	LF73	87
11	BD9 XIX	1.0	12.5	0.5	0.5	10.5	LF72	LF73	87
12	BD9 XII	1.0	12.5	0.5	0.5	10.5	LF72	LF73	87
13	BD9 IX*	1.0	12.5	0.5	0.5	10.5	LF72	LF73	87
14	BD9 XIV	1.0	12.5	0.5	0.5	10.5	LF72	LF73	87
15	BD6 XI*	1.0	12.5	0.5	0.5	10.5	LF72	LF73	87
16	BD6 VI*	1.0	12.5	0.5	0.5	10.5	LF72	LF73	87
17	BD6 X	1.0	12.5	0.5	0.5	10.5	LF72	LF73	87
18	BD6 XV*	1.0	12.5	0.5	0.5	10.5	LF72	LF73	87
19	BD6 XIV*	1.0	12.5	0.5	0.5	10.5	LF72	LF73	87
20	BD8 IX	1.0	12.5	0.5	0.5	10.5	LF72	LF73	87
21	BD8 II*	1.0	12.5	0.5	0.5	10.5	LF72	LF73	87
22	BD8 III	1.0	12.5	0.5	0.5	10.5	LF72	LF73	87
23	BD7 XVIII	1.0	12.5	0.5	0.5	10.5	LF72	LF73	87
24	BD7 I	1.0	12.5	0.5	0.5	10.5	LF72	LF73	87
25	BD7 II	1.0	12.5	0.5	0.5	10.5	LF72	LF73	87
26	BD1 VII*	1.0	12.5	0.5	0.5	10.5	LF72	LF73	87
27	BD1 III	1.0	12.5	0.5	0.5	10.5	LF72	LF73	87
28	BD1 XV	1.0	12.5	0.5	0.5	10.5	LF72	LF73	87
29	BD1 X	1.0	12.5	0.5	0.5	10.5	LF72	LF73	87
30	NTC	0.0	12.5	0.5	0.5	11.5	LF72	LF73	87

Thermal Cycling conditions:	Initial Denaturation	Denaturation	Annealing	Extension	Final Extension
	95C for 2mins	95C for 0.5mins	60C for 1min	68C for 0.5mins	68C for 5mins
			30 cycles		

Figure S7b. Full length gel for the results shown in **Figure S3a** of the RT-PCR analysis of *ZmMYB167* transgenic *Brachypodium* using *S-adenosylmethionine decarboxylase* gene-specific primers. * Denotes null-segregant (NS). NTC, no template control.

ZmMYB167 transgene

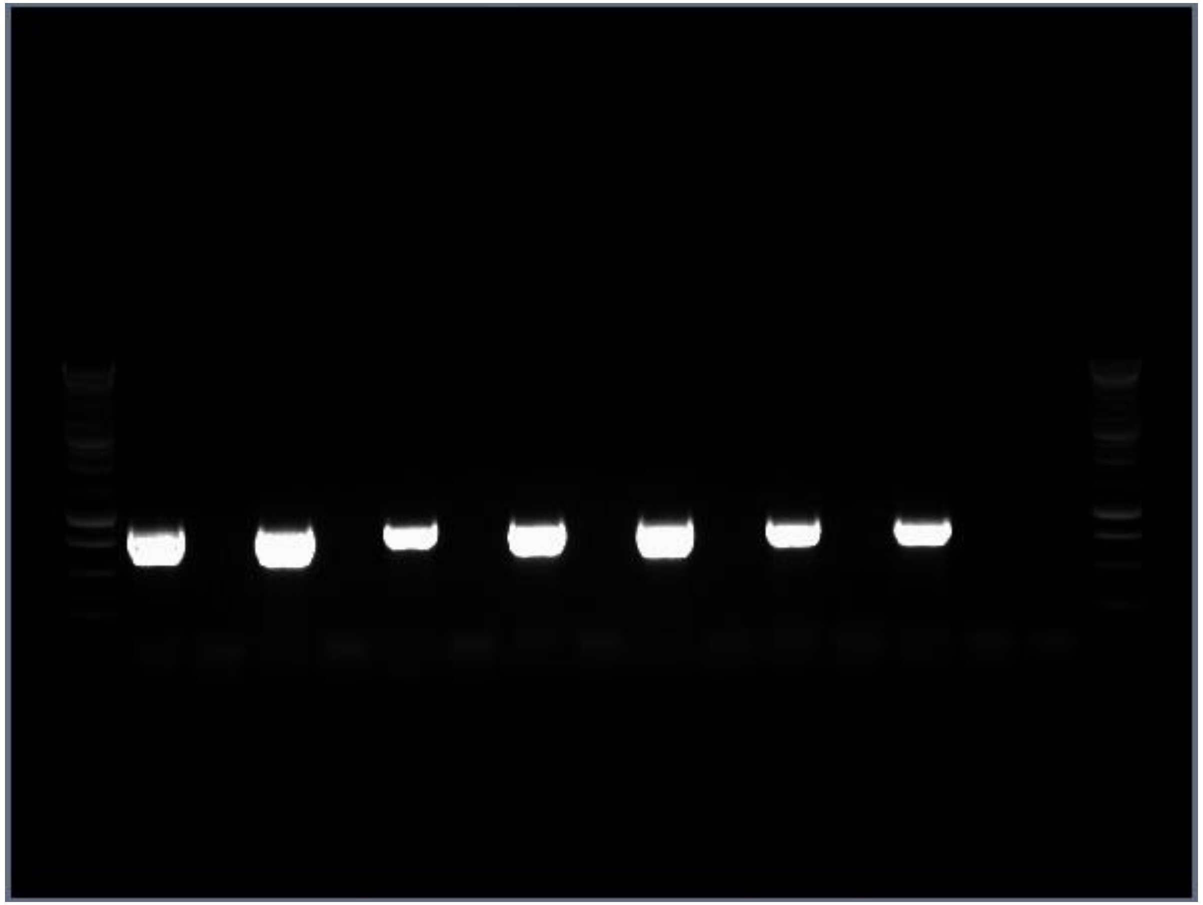

PCR Lane No	gDNA template F1 ZmMYB167 line	gDNA template (~100ng/µl)	5X Green Go Taq Reaction Buffer (µl)	10mM dNTPs (µl)	Go Taq DNA polymerase (µl)	Forward Primer 10µM (µl)	Reverse Primer 10µM (µl)	DEPC water (µl)	Forward Primer	Reverse Primer	Amplicon size (bp)
1	1kb molecular marker										
2	Zm1	3	5	0.5	0.125	0.5	0.5	15.4	MB406	MB350	870
3	Zm1 NS	3	5	0.5	0.125	0.5	0.5	15.4	MB406	MB350	870
4	Zm2	3	5	0.5	0.125	0.5	0.5	15.4	MB406	MB350	870
5	Zm2 NS	3	5	0.5	0.125	0.5	0.5	15.4	MB406	MB350	870
6	Zm3	3	5	0.5	0.125	0.5	0.5	15.4	MB406	MB350	870
7	Zm3 NS	3	5	0.5	0.125	0.5	0.5	15.4	MB406	MB350	870
8	Zm4	3	5	0.5	0.125	0.5	0.5	15.4	MB406	MB350	870
9	Zm4 NS	3	5	0.5	0.125	0.5	0.5	15.4	MB406	MB350	870
10	Zm5	3	5	0.5	0.125	0.5	0.5	15.4	MB406	MB350	870
11	Zm5 NS	3	5	0.5	0.125	0.5	0.5	15.4	MB406	MB350	870
12	Zm6	3	5	0.5	0.125	0.5	0.5	15.4	MB406	MB350	870
13	Zm6 NS	3	5	0.5	0.125	0.5	0.5	15.4	MB406	MB350	870
14	Zm7	3	5	0.5	0.125	0.5	0.5	15.4	MB406	MB350	870
15	Zm7 NS	3	5	0.5	0.125	0.5	0.5	15.4	MB406	MB350	870
16	NTC	0	5	0.5	0.125	0.5	0.5	18.4	MB406	MB350	870

Thermal Cycling conditions:	Initial Denaturation	Denaturation	Annealing	Extension	Final Extension
	**95C for 2mins**	**95C for 0.5mins**	**55C for 1min**	**72C for 1mins**	**72C for 5mins**
			**35 cycles**		

Figure S7c. Full length gel for the results shown in **Figure 3b** of the genomic DNA PCR analysis of *ZmMYB167* transgenic and corresponding null-segregant (NS) plants using primers covering the *ZmUbI1* promoter region and the *ZmMYB167* transgene-specific region. NS, null-segregant control; NTC, no template control.

5’ UTR/endogenous ZmMYB167 and *Peptidase C14* transcript

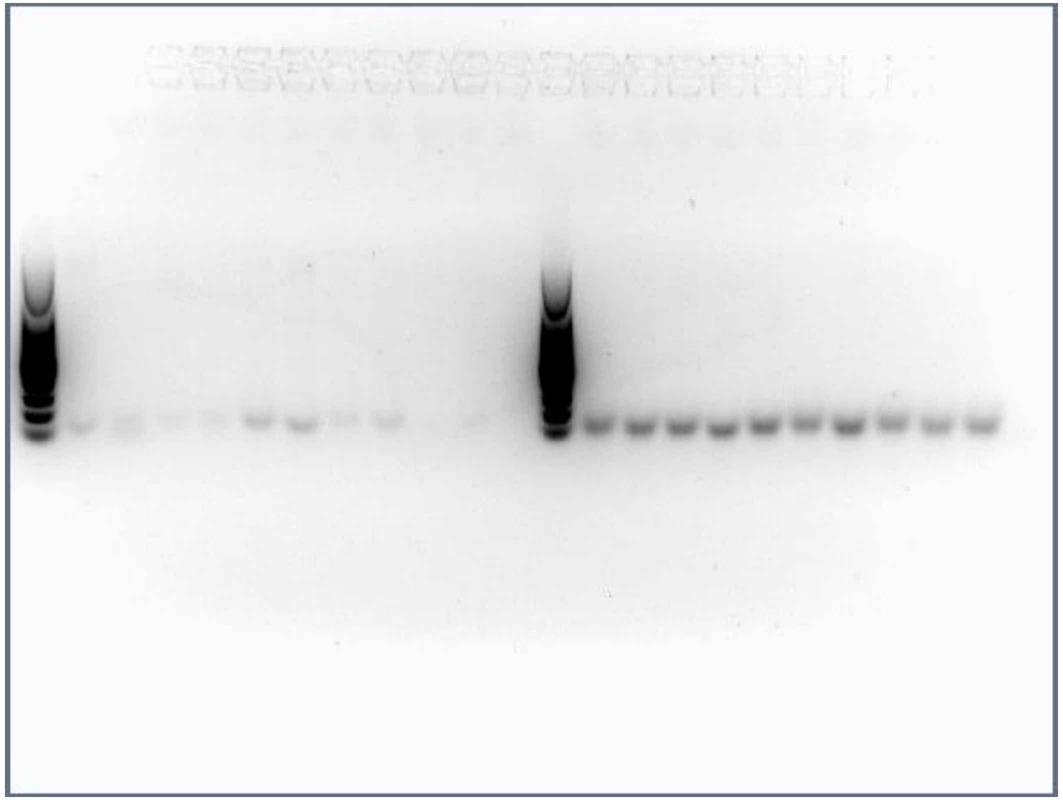

PCR Lane No	cDNA template F1 ZmMYB167 line	cDNA template (~50ng) (µl)	Forward Primer 5µM (µl)	Reverse Primer 5µM (µl)	Quick-Load® Taq 2X Master Mix (µl)	DEPC water (µl)	Forward Primer	Reverse Primer	Amplicon size (bp)
1	100bp molecular marker								
2	Zm1	1.0	0.5	0.5	12.5	10.5	RB3 5'UTR ZmMYB167	RB4 5'UTR ZmMYB167	108
3	Zm1 NS	1.0	0.5	0.5	12.5	10.5	RB3 5'UTR ZmMYB167	RB4 5'UTR ZmMYB167	108
4	Zm2	1.0	0.5	0.5	12.5	10.5	RB3 5'UTR ZmMYB167	RB4 5'UTR ZmMYB167	108
5	Zm2 NS	1.0	0.5	0.5	12.5	10.5	RB3 5'UTR ZmMYB167	RB4 5'UTR ZmMYB167	108
6	Zm3	1.0	0.5	0.5	12.5	10.5	RB3 5'UTR ZmMYB167	RB4 5'UTR ZmMYB167	108
7	Zm3 NS	1.0	0.5	0.5	12.5	10.5	RB3 5'UTR ZmMYB167	RB4 5'UTR ZmMYB167	108
8	Zm4	1.0	0.5	0.5	12.5	10.5	RB3 5'UTR ZmMYB167	RB4 5'UTR ZmMYB167	108
9	Zm4 NS	1.0	0.5	0.5	12.5	10.5	RB3 5'UTR ZmMYB167	RB4 5'UTR ZmMYB167	108
10	Zm5	1.0	0.5	0.5	12.5	10.5	RB3 5'UTR ZmMYB167	RB4 5'UTR ZmMYB167	108
11	Zm5 NS	1.0	0.5	0.5	12.5	10.5	RB3 5'UTR ZmMYB167	RB4 5'UTR ZmMYB167	108
12	NTC	0.0	0.5	0.5	12.5	11.5	RB3 5'UTR ZmMYB167	RB4 5'UTR ZmMYB167	108
13	100bp molecular marker								
14	Zm1	1.0	0.5	0.5	12.5	10.5	MB185	MB186	81
15	Zm1 NS	1.0	0.5	0.5	12.5	10.5	MB185	MB186	81
16	Zm2	1.0	0.5	0.5	12.5	10.5	MB185	MB186	81
17	Zm2 NS	1.0	0.5	0.5	12.5	10.5	MB185	MB186	81
18	Zm3	1.0	0.5	0.5	12.5	10.5	MB185	MB186	81
19	Zm3 NS	1.0	0.5	0.5	12.5	10.5	MB185	MB186	81
20	Zm4	1.0	0.5	0.5	12.5	10.5	MB185	MB186	81
21	Zm4 NS	1.0	0.5	0.5	12.5	10.5	MB185	MB186	81
22	Zm5	1.0	0.5	0.5	12.5	10.5	MB185	MB186	81
23	Zm5 NS	1.0	0.5	0.5	12.5	10.5	MB185	MB186	81
24	NTC	0.0	0.5	0.5	12.5	11.5	MB185	MB186	81

		30 cycles				
**Thermal Cycling conditions:**	**Initial Denaturation**	**Denaturation**	**Annealing**	**Extension**	**Final Extension**	**Hold**
	**95C for 2mins**	**95C for 0.5mins**	**60C for 1min**	**68C for 0.5mins**	**68C for 5mins**	**4C**

Figure S7d. Full length gel for the results shown in **Figure 5a** of the Semi-quantitative RT-PCR analysis of endogenous *ZmMYB167* gene of transgenic *ZmMYB167* maize plants. *Peptidase C14* was used as a reference gene. NS, null-segregant control; NTC, no template control. Image was colour inverted for better visibility.

ZmMYB17 transcript

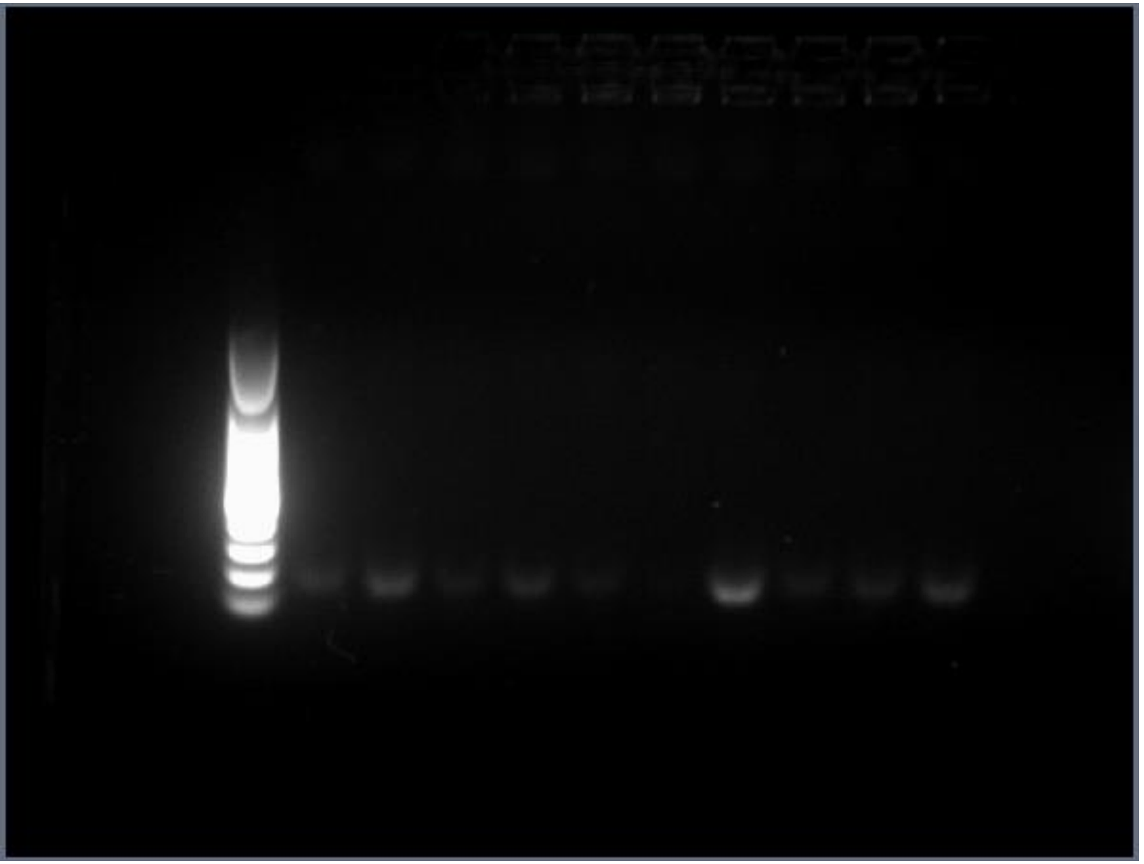

PCR Lane No	cDNA template F1 ZmMYB167 line	cDNA template (~50ng)(µl)	Forward Primer 5µM (µl)	Reverse Primer 5µM (µl)	Quick-Load® Taq 2X Master Mix (µl)	DEPC water (µl)	Forward Primer	Reverse Primer	Amplicon size (bp)
1	100bp molecular marker								
2	Zm1	1.0	0.5	0.5	12.5	10.5	RB5 ZmMYB17	RB6 ZmMYB17	115
3	Zm1 NS	1.0	0.5	0.5	12.5	10.5	RB5 ZmMYB17	RB6 ZmMYB17	115
4	Zm2	1.0	0.5	0.5	12.5	10.5	RB5 ZmMYB17	RB6 ZmMYB17	115
5	Zm2 NS	1.0	0.5	0.5	12.5	10.5	RB5 ZmMYB17	RB6 ZmMYB17	115
6	Zm3	1.0	0.5	0.5	12.5	10.5	RB5 ZmMYB17	RB6 ZmMYB17	115
7	Zm3 NS	1.0	0.5	0.5	12.5	10.5	RB5 ZmMYB17	RB6 ZmMYB17	115
8	Zm4	1.0	0.5	0.5	12.5	10.5	RB5 ZmMYB17	RB6 ZmMYB17	115
9	Zm4 NS	1.0	0.5	0.5	12.5	10.5	RB5 ZmMYB17	RB6 ZmMYB17	115
10	Zm5	1.0	0.5	0.5	12.5	10.5	RB5 ZmMYB17	RB6 ZmMYB17	115
11	Zm5 NS	1.0	0.5	0.5	12.5	10.5	RB5 ZmMYB17	RB6 ZmMYB17	115
12	NTC	0.0	0.5	0.5	12.5	11.5	RB5 ZmMYB17	RB6 ZmMYB17	115

		35 cycles				
**Thermal Cycling conditions:**	**Initial Denaturation**	**Denaturation**	**Annealing**	**Extension**	**Final Extension**	**Hold**
	**95C for 2mins**	**95C for 0.5mins**	**60C for 1min**	**68C for 0.5mins**	**68C for 5mins**	**4C**

Figure S7e. Full length gel for the results shown in **Figure 5b** of the semi-quantitative RT-PCR analysis of the *ZmMYB17* gene of transgenic *ZmMYB167* maize plants. *Peptidase C14* (**see Figure S7f**) was used as a reference gene. NS, null-segregant control; NTC, no template control.

Peptidase C14 transcript

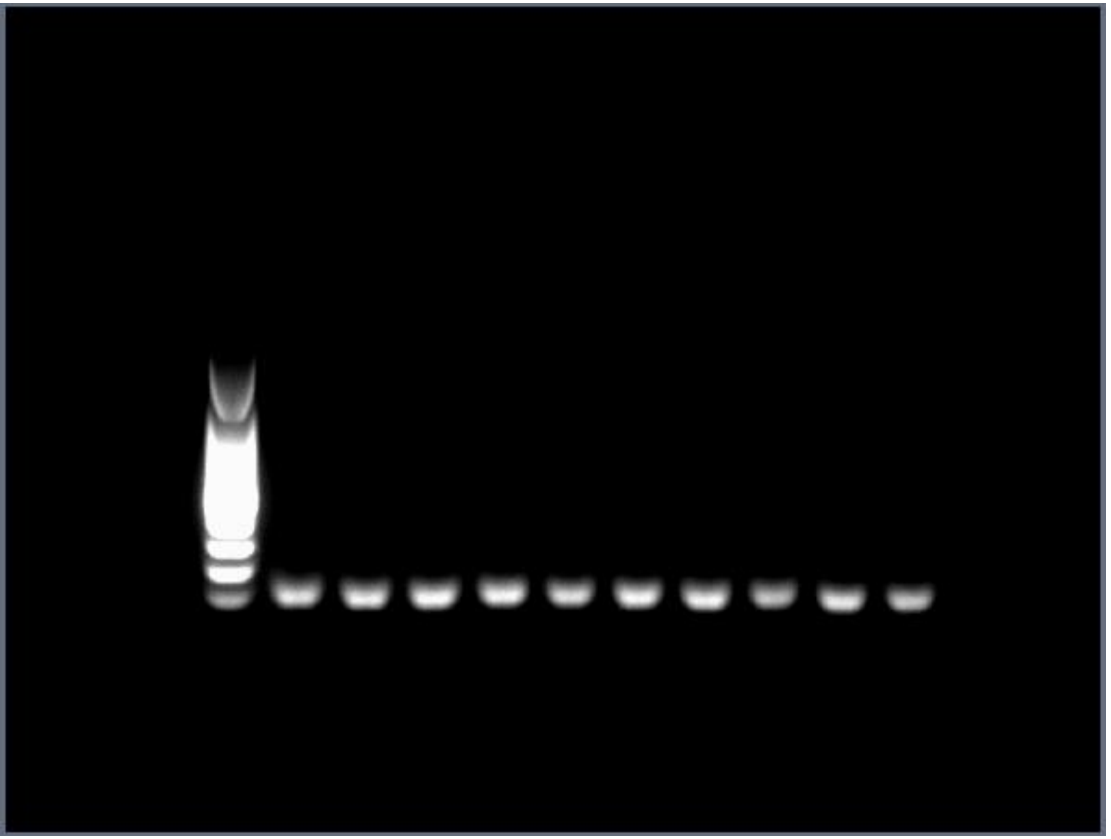

PCR Lane No	cDNA template F1 ZmMYB167 line	cDNA template (~50ng)(µl)	Forward Primer 5µM (µl)	Reverse Primer 5µM (µl)	Quick-Load® Taq 2X Master Mix (µl)	DEPC water (µl)	Forward Primer	Reverse Primer	Amplicon size (bp)
1	100bp molecular marker								
2	Zm1	1.0	0.5	0.5	12.5	10.5	MB185	MB186	81
3	Zm1 NS	1.0	0.5	0.5	12.5	10.5	MB185	MB186	81
4	Zm2	1.0	0.5	0.5	12.5	10.5	MB185	MB186	81
5	Zm2 NS	1.0	0.5	0.5	12.5	10.5	MB185	MB186	81
6	Zm3	1.0	0.5	0.5	12.5	10.5	MB185	MB186	81
7	Zm3 NS	1.0	0.5	0.5	12.5	10.5	MB185	MB186	81
8	Zm4	1.0	0.5	0.5	12.5	10.5	MB185	MB186	81
9	Zm4 NS	1.0	0.5	0.5	12.5	10.5	MB185	MB186	81
10	Zm5	1.0	0.5	0.5	12.5	10.5	MB185	MB186	81
11	Zm5 NS	1.0	0.5	0.5	12.5	10.5	MB185	MB186	81
12	NTC	0.0	0.5	0.5	12.5	11.5	MB185	MB186	81

		35 cycles				
**Thermal Cycling conditions:**	**Initial Denaturation**	**Denaturation**	**Annealing**	**Extension**	**Final Extension**	**Hold**
	**95C for 2mins**	**95C for 0.5mins**	**60C for 1min**	**68C for 0.5mins**	**68C for 5mins**	**4C**

Figure S7f. Full length gel for the results shown in **Figure 5b** of *Peptidase C14* used as a reference gene for the semi-quantitative RT-PCR analysis. NS, null-segregant control; NTC, no template control.

Genotype	Plant height (cm)	Flowering time (days)	Stem biomass yield (g)
**NS Zm1**	178.0 ± 2.5	85 ± 0.7	43.2 ± 2.4
**Zm1**	172.5 ± 3.8	84 ± 0.0	37.6 ± 4.8
**NS Zm2**	173.5 ± 1.5	83 ± 1.5	47.2 ± 0.6
**Zm2**	177.0 ± 7.0	83 ± 1.5	53.8 ± 4.5
**NS Zm3**	196.9 ± 7.7	86 ± 0.3	40.8 ± 3.7
**Zm3**	185.6 ± 8.1	87 ± 0.9	39.8 ± 3.2
**NS Zm4**	198.2 ± 11.7	79 ± 1.2	40.3 ± 3.0
**Zm4**	189.3 ± 14.9	81 ± 2.0	38.2 ± 6.3
**NS Zm5**	134.9 ± 6.3	86 ± 0.3	29.1 ± 1.6
**Zm5**	124.1 ± 6.0	87 ± 1.0	24.5 ± 3.4

Table S1. Agronomic measurements of transgenic *ZmMYB167* maize plants. Plant height (cm) at VT stage was from node 7 to the V18 leaf collar and the dry biomass weight was obtained from above ground stem biomass harvested at VT stage. Data are means ± SE of at least three transgenic and null-segregant (NS) plants (n≥3). Student's *t*-test: *P ≤0.05; **P ≤0.01.

Genotype	Extractives-free basis					
	% Glucan	% Xylan	% Mannan	% Arabinan	% Galactan	% Klason Lignin
**NS Zm1**	48.2 ± 0.2	29.4 ± 0.4	2.2 ± 0.1	3.8 ± 0.1	1.5 ± 0.0	14.9 ± 0.2
**Zm1**	47.2 ± 0.7	29.7 ± 0.6	1.9 ± 0.1	3.6 ± 0.1	1.5 ± 0.2	16.1 ± 0.3*
**NS Zm2**	47.8 ± 0.6	29.3 ± 0.4	1.7 ± 0.2	3.8 ± 0.1	1.2 ± 0.2	16.3 ± 0.3
**Zm2**	47.5 ± 0.6	28.8 ± 0.3	1.6 ± 0.0	3.4 ± 0.0*	1.3 ± 0.1	17.5 ± 0.4**
**NS Zm3**	50.9 ± 0.2	28.2 ± 0.5	1.8 ± 0.1	3.7 ± 0.0	1.4 ± 0.1	14.1 ± 0.4
**Zm3**	48.5 ± 0.6**	28.6 ± 0.2	1.6 ± 0.1*	3.9 ± 0.2	1.4 ± 0.1	15.9 ± 0.6**
**NS Zm4**	49.1 ± 0.3	28.2 ± 0.7	1.7 ± 0.0	3.5 ± 0.2	1.2 ± 0.1	16.3 ± 0.5
**Zm4**	46.6 ± 0.3*	29.9 ± 0.5	1.9 ± 0.0	3.4 ± 0.1	1.2 ± 0.1	17.0 ± 0.1
**NS Zm5**	47.3 ± 0.6	30.9 ± 0.4	1.8 ± 0.4	4.9 ± 0.3	1.9 ± 0.1	13.3 ± 0.2
**Zm5**	47.2 ± 0.7	30.5 ± 0.4	1.5 ± 0.2	4.3 ± 0.1	1.7 ± 0.1*	14.9 ± 0.4*

Table S2. Structural carbohydrates and lignin in transgenic *ZmMYB167* maize plants. Data are means ± SE of three technical replicates from one individual plant per event. Lignin is total lignin (acid soluble and acid insoluble lignin). Ash, acetyl and extractives content were not determined. Data was normalized to a summative mass closure of 100%. NS, null-segregant. Student's *t*-test: *$P \leq 0.05$; **$P \leq 0.01$.

Table S3. Primers used for DNA sequencing and genomic DNA PCR analysis.

Target template	Accession number	Primer name	Sequence of gene-specific primer
ZmUbi1 promoter	EU161568	MB350	F:5‘-TGCAGCATCTATTCATATGCTCT-3‘
35S terminator		MB348	F:5‘-GCAGGTCACTGGATTTTGGT-3‘
ZmMYB167 exon	GRMZM2G037650; Zm00001d032032	MB403	F:5‘-TGAGCAACGGCAATGAGCTT-3‘
		MB404	R:5‘-ACCAAGTTGGAGCTGCACATG-3‘
		MB405	F:5‘-CCATCATAGGGAGGTTCCGC-3‘
		MB406	R:5‘-CCACTGACAAGTCGGTCTCC-3‘

Table S4. Primers used for RT-PCR and Real-time PCR.

Gene	Accession number/ Contig number	Sequence of gene-specific primers	Amplicon size (bp)
ZmMYB167	GRMZM2G037650; Zm00001d032032	F:5‘-CGGAACAACAGACTGGCTGC-3‘	84
		R:5‘-GAGGCGCGGAACATTGAAT-3‘	
5‘UTR/endogenous ZmMYB167	GRMZM2G037650; Zm00001d032032	F:5‘-GTACAAGTTGCCAGCTGATAGTAA-3‘	108
		R:5‘-TGAGCTTCTTGTCCTCCTCCG-3‘	
ZmMYB17	GRMZM2G138427; Zm00001d053210	F:5‘-GTGCTTAATCTAGTACAAGCTGCC-3‘	115
		R:5‘-TTCTTGTCCTCCTCCGCTGT-3‘	
Peptidase C14	MZ00027363	F:5‘-CGTGATGAAAGCACGTGAGCT-3‘	81
		R:5‘-TGAACATGCTCATCGCTGC-3‘	
SamDC	Bradi5g14640	F:5‘-ATCCATGTGACCCCTGAGGA-3‘	87
		R:5‘-CCTCTTGACAAGGTCGCCAT-3‘	

Table S5. Information on the standard curves for Real-time PCR. A standard curve with a PCR efficiency of “2” doubles an amplified product at each cycle. PCR efficiency for each primer-set was roughly equal and a value of ≥1.8 was accepted for valid comparisons between different samples. Data are means ± SE of two experiments.

Gene	Final primer conc. Fw/Rv (nM)	Cp range	Slope	Y-Interception	LC480 PCR efficiency
ZmMYB167	50/900	20-23	-3.42 ± 0.07	29.95 ± 0.37	1.96 ± 0.03
Peptidase C14	900/900	20-23	-3.55 ± 0.17	28.03 ± 0.08	1.92 ± 0.06
